# Conservation and Variability of Dengue Virus Proteins: Implications for Vaccine Design

**DOI:** 10.1371/journal.pntd.0000272

**Published:** 2008-08-13

**Authors:** Asif M. Khan, Olivo Miotto, Eduardo J. M. Nascimento, K. N. Srinivasan, A. T. Heiny, Guang Lan Zhang, E. T. Marques, Tin Wee Tan, Vladimir Brusic, Jerome Salmon, J. Thomas August

**Affiliations:** 1 Department of Biochemistry, Yong Loo Lin School of Medicine, National University of Singapore, Singapore; 2 Institute of Systems Science, National University of Singapore, Singapore; 3 Department of Medicine, Division of Infectious Diseases, The Johns Hopkins University School of Medicine, Baltimore, Maryland, United States of America; 4 Department of Pharmacology and Molecular Sciences, The Johns Hopkins University School of Medicine, Baltimore, Maryland, United States of America; 5 Product Evaluation and Registration Division, Centre for Drug Administration, Health Sciences Authority, Singapore; 6 Cancer Vaccine Center, Dana-Farber Cancer Institute, Boston, Massachusetts, United States of America; University of California Berkeley, United States of America

## Abstract

**Background:**

Genetic variation and rapid evolution are hallmarks of RNA viruses, the result of high mutation rates in RNA replication and selection of mutants that enhance viral adaptation, including the escape from host immune responses. Variability is uneven across the genome because mutations resulting in a deleterious effect on viral fitness are restricted. RNA viruses are thus marked by protein sites permissive to multiple mutations and sites critical to viral structure-function that are evolutionarily robust and highly conserved. Identification and characterization of the historical dynamics of the conserved sites have relevance to multiple applications, including potential targets for diagnosis, and prophylactic and therapeutic purposes.

**Methodology/Principal Findings:**

We describe a large-scale identification and analysis of evolutionarily highly conserved amino acid sequences of the entire dengue virus (DENV) proteome, with a focus on sequences of 9 amino acids or more, and thus immune-relevant as potential T-cell determinants. DENV protein sequence data were collected from the NCBI Entrez protein database in 2005 (9,512 sequences) and again in 2007 (12,404 sequences). Forty-four (44) sequences (pan-DENV sequences), mainly those of nonstructural proteins and representing ∼15% of the DENV polyprotein length, were identical in 80% or more of all recorded DENV sequences. Of these 44 sequences, 34 (∼77%) were present in ≥95% of sequences of each DENV type, and 27 (∼61%) were conserved in other *Flaviviruses*. The frequencies of variants of the pan-DENV sequences were low (0 to ∼5%), as compared to variant frequencies of ∼60 to ∼85% in the non pan-DENV sequence regions. We further showed that the majority of the conserved sequences were immunologically relevant: 34 contained numerous predicted human leukocyte antigen (HLA) supertype-restricted peptide sequences, and 26 contained T-cell determinants identified by studies with HLA-transgenic mice and/or reported to be immunogenic in humans.

**Conclusions/Significance:**

Forty-four (44) pan-DENV sequences of at least 9 amino acids were highly conserved and identical in 80% or more of all recorded DENV sequences, and the majority were found to be immune-relevant by their correspondence to known or putative HLA-restricted T-cell determinants. The conservation of these sequences through the entire recorded DENV genetic history supports their possible value for diagnosis, prophylactic and/or therapeutic applications. The combination of bioinformatics and experimental approaches applied herein provides a framework for large-scale and systematic analysis of conserved and variable sequences of other pathogens, in particular, for rapidly mutating viruses, such as influenza A virus and HIV.

## Author Summary

Dengue viruses (DENVs) circulate in nature as a population of 4 distinct types, each with multiple genotypes and variants, and represent an increasing global public health issue with no prophylactic and therapeutic formulations currently available. Viral genomes contain sites that are evolutionarily stable and therefore highly conserved, presumably because changes in these sites have deleterious effects on viral fitness and survival. The identification and characterization of the historical dynamics of these sites in DENV have relevance to several applications such as diagnosis and drug and vaccine development. In this study, we have identified sequence fragments that were conserved across the majority of available DENV sequences, analyzed their historical dynamics, and evaluated their relevance as candidate vaccine targets, using various bioinformatics-based methods and immune assay in human leukocyte antigen (HLA) transgenic mice. This approach provides a framework for large-scale and systematic analysis of other human pathogens.

## Introduction

Dengue viruses (DENVs) are mosquito-borne pathogens of the family *Flaviviridae*, genus *Flavivirus,* which are phylogenetically related to other important human pathogens, such as *Yellow fever* (YFV), *Japanese encephalitis* (JEV), and *West Nile* (WNV) viruses, among others. DENVs are enveloped, single-stranded RNA (+) viruses coding for a polyprotein precursor of approximately 3,400 amino acids, which is cleaved into three structural (capsid, C; precursor membrane and membrane, prM/M; envelope, E) and seven nonstructural proteins (NS1, 2a, 2b, 3, 4a, 4b and 5). Viral replication occurs in the cytoplasm in association with virus-induced membrane structures and involves the NS proteins. There are 4 genetically distinct DENV types, referred to as DENV-1 to -4, with multiple genotypic variants [1],[2]. DENVs are transmitted to humans primarily by *Aedes aegypti* mosquitoes and cause a wide range of symptoms from an unapparent or mild dengue fever (DF) to severe dengue hemorrhagic fever (DHF)/dengue shock syndrome (DSS) that may be fatal. It is estimated that more than 100 million people are infected each year, with up to several hundred thousand DHF/DSS cases [3]. To date, there is no licensed prophylactic vaccine and no specific therapeutic formulation available.

Adaptive immune responses include cellular responses to short peptides derived from self and foreign proteins by proteolysis. The peptides are presented to T-cell receptors (TCRs) by major histocompatibility complex (MHC) molecules, referred to as human leukocyte antigen (HLA) molecules in humans. HLA class I and class II molecules bind and present peptides to CD8 and CD4 T-cells, respectively, that play a critical role in antigen (Ag)-specific cytotoxic responses and the induction and maintenance of Ag-specific memory responses [4]–[6]. Peptides that are recognized by the T cells and trigger an immune response are referred to as T-cell determinants. One problem in developing a tetravalent DENV vaccine is the viral diversity [7], with rather low intra-type, but high inter-type variability, resulting in type-specific and type cross-reactive T-cell determinants [8]. This variability of related structures gives rise to a large number of variant peptide sequences with one or more amino acid differences that may function as alternative determinants, or altered peptide ligands [9], and affect anti-DENV host immunity [10],[11]. There is abundant evidence that interactions of memory T cells with peptide ligands bearing amino acid substitutions at TCR contact residues may alter T-cell activation and effector function [9], [12]–[15]. Even a single amino acid substitution can impair the function of T cells in a variety of ways, producing profoundly different phenotypes that range from modified stimulatory function to complete inhibition [14]. These findings suggest that infection or immunization with multiple DENV types, as is the case with some tetravalent vaccines, may lead to T-cell responses to variant peptides that might be deleterious. There is also the possibility that the altered-ligand phenomenon and cross-reactive T-cell responses, referred to as original antigenic sin, may play a role in DHF/DSS [7],[11],[16],[17]. Although the etiology of DHF and DSS is only partially understood, this consideration may have profound implications for the safety and efficiency of candidate vaccines.

The objective of this study was to search for sequence regions conserved across the majority of DENVs and representing potential immune targets [18]. Bioinformatics-based approaches were used to (a) extract all DENV sequences available in public databases, (b) identify and examine the structure-function relationship and distribution in nature of sequences that are highly conserved in the majority of DENVs (referred to as pan-DENV sequences), (c) analyze the variability of DENV sequences, and (d) examine the immune relevance of the conserved sequences as potential T-cell determinants that would be applicable to the majority of the human population worldwide [19]. We have also correlated the conserved DENV sequences to previously reported T-cell determinants and further identified novel candidate T-cell determinants by analyzing HLA-restricted immune responses in HLA transgenic mice.

## Methods

### Methodology overview

**Figure 1:**
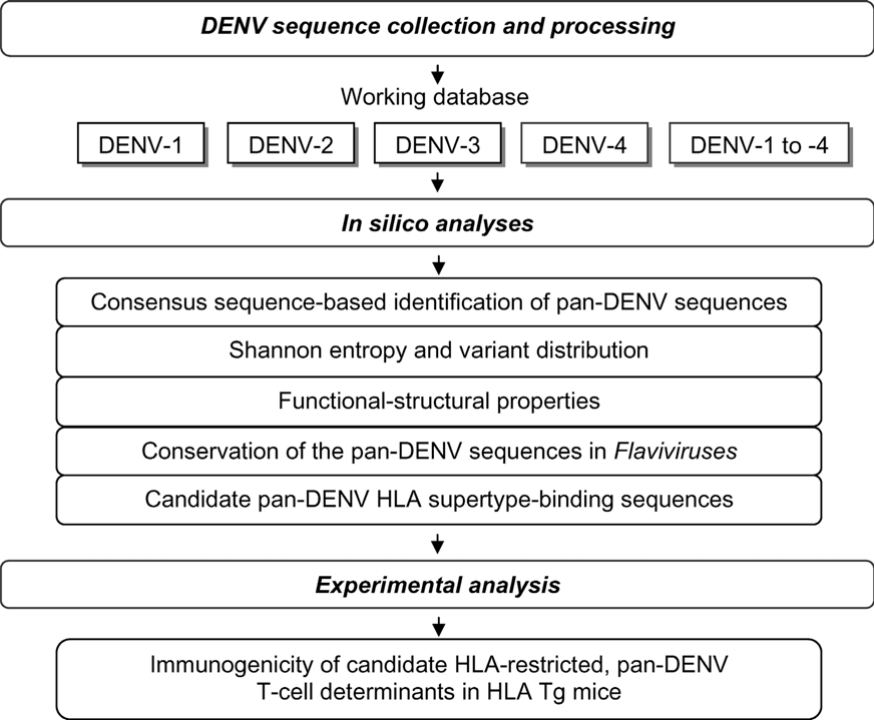
Overview of the bioinformatics and experimental approaches employed for the identification and analysis of the pan-DENV sequences.

The bioinformatics approaches and rationale for the methodology adopted in this study have been previously described [20] and are summarized in **Figure 1**.

### Data collection and sequence organization

DENV protein sequences were retrieved from the NCBI Entrez protein database in December 2005, and again in December 2007 for validation purposes, by use of a taxonomy ID search via the NCBI taxonomy browser [21]. The taxonomy IDs for DENV-1 to -4 were 11053, 11060, 11069 and 11070, respectively. The data for 2007 were processed separately from the 2005 dataset, but using identical procedures.

The sequences of the DENV proteins C, prM, E, NS1, NS2a, NS2b, NS3, NS4a, NS4b and NS5 were extracted from the database records (**Dataset S1**) by multiple sequence alignments, and application of the known cleavage sites obtained from the annotation of the GenPept [21] reference polyprotein sequences of DENV-1 to -4 (AAF59976, P14340, AAM51537, AAG45437, respectively), and from the literature [22]. Grouping of the sequences of each DENV type was performed by BLAST [23] followed by CLUSTALX 1.83 [24] multiple sequence alignments. Both full-length and partial sequences of each DENV protein were used for analysis, and identical sequences were not removed from datasets, unless otherwise indicated. All multiple sequence alignments were manually inspected and corrected for misalignments.

### Identification of pan-DENV sequences

The DENV protein sequences were examined by a consensus-sequence based approach [25] to identify sequence fragments that were common across the 4 types. The consensus sequences for the proteins of each type (intra-type consensus) were first derived by multiple sequence alignments to select the predominant residue at each amino acid position. The 4 intra-type consensus sequences for a given protein (one from each type) were then aligned to reveal sequence fragments identical across each of the types that were at least 9 amino acids long. This minimum length was chosen because it represents the binding core length of a majority of HLA-restricted T-cell determinants [26]. Only sequence fragments that were identical in at least 80% of the sequences of each of the 4 types were retained for further analyses. Peptides with residue X in the alignment were ignored from the percentage representation (*i.e.* frequency) computation. The 80% intra-type representation cut-off was chosen because 44 of the 46 sequence fragments that were common across the 4 DENV types exhibited intra-type representation of ≥81%, and those two that did not had significantly lower representation (∼56–67%) in one of the 4 types.

### Information entropy analysis of pan-DENV sequences

Shannon information entropy [20],[27] was used to study the diversity of DENV protein sequences within each type (intra-type diversity) and across all DENVs (pan-DENV diversity) and to assess the predicted evolutionary stability of the identified pan-DENV sequences. All entropy analyses were carried out by using the in-house developed Antigenic Variability Analyser tool (AVANA) [28]. For immunological applications, the entropy measure for antigenic sequences was based on nonamer peptides [26], centered at any given position in the alignment. Applying Shannon's formula, the nonamer peptide entropy *H(x)* at any given position x in the alignment was computed by
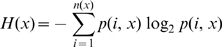
where *p(i, x)* is the probability of a particular nonamer peptide *i* being centered at position *x*. The entropy value increases with *n(x)*, the total number of peptides observed at position *x*; it is also sensitive to the relative frequency of the peptides; such that it decreases when one peptide is clearly dominant (*i.e.* the position is conserved). Only sequences that contain a valid amino acid at position *x* were used for the entropy computation, and the alignment gaps were ignored. Although gaps tend to occur in high-diversity regions, proteins that have a high fraction of gaps have reduced statistical support, yielding an artificially low entropy value; for this reason, positions where more than 50% of sequences contained a gap were discarded. Because of the statistical nature of the entropy measure, both complete protein and shorter fragment sequences were used in this computation. The first and last 4 positions in the alignment of each protein were not assigned any peptide entropy value as they cannot be the center of a nonamer.

In theory, nonamer entropy values can range from 0, for a completely conserved nonamer peptide in all sequences analyzed, to 39 (log_2_ 20^9^); in practice, however, the upper bound is very much lower for alignments of closely related sequences. For finite-size sets of sequences, entropy computations are affected by the sequence count in the alignment. For an alignment of N sequences, alignment size bias is proportional to 1/N [29]. This relationship allows a correction for size bias by applying to each alignment a statistical adjustment that estimates entropy values for an infinitely-sized alignment with analogous peptide distribution. To obtain such an estimate, the alignment was repeatedly randomly sampled to create smaller alignments of varying size, whose entropy was measured. At each alignment position, the entropy of these subset alignments of size N was plotted against 1/N, using a linear regression to extrapolate the entropy estimate for N→∞. The regression's coefficient of determination (r^2^) was used as a goodness-of-fit of the resulting estimate. In this study, size bias correction was applied to all entropy calculations, so that alignment sequence counts could be ignored in comparisons. All entropy values reported are therefore infinite-size set estimates, rather than the values directly computed from the alignments.

### Nonamer variant analysis of pan-DENV sequences

Data from information entropy analysis were used to study the distribution of the representation of nonamer variant peptides in DENV sequences, within and across the types. For any given position *x* in the alignment, the combined representation of all nonamers, excluding the predominant peptide, was computed. The predominant nonamer was the peptide that was contained in the majority of the sequences at the position in the alignment. All the other peptides that differed by at least one amino acid from the predominant nonamer were defined as variants.

### Functional and structural analyses of pan-DENV sequences

The known and putative structural and functional properties of pan-DENV sequences were searched in the literature and by use of the Prosite [30], via ScanProsite [31], and Pfam [32] databases. When possible, the sequences were mapped on the three-dimensional (3-D) structures of available DENV Ag in the PDB database [33] by use of ICM-Browser version 3.3 (www.molsoft.com). X-ray diffraction 3-D structures were visualized by use of the Corey, Pauling and Koltun (cpk) representation in the ICM-Browser.

### Identification of pan-DENV sequences common to other viruses and organisms

Pan-DENV sequences that overlapped at least 9 consecutive amino acid sequences of other viruses and organisms were identified by performing BLAST search against viral protein sequences reported at NCBI (as of July 2007), excluding DENV sequences (parameters set: limit by Entrez query “txid10239[Organism:exp] NOT txid12637[Organism:exp]”; automatically adjust parameters for short sequences option enabled; low-complexity filter disabled; alignments: 20,000), and against protein sequences of all organisms excluding viruses (parameters set: limit by Entrez query “Root[ORGN] NOT Viruses[ORGN] NOT txid81077[ORGN]”; automatically adjust parameters for short sequences option enabled; low-complexity filter disabled; alignments: 20,000). The keyword “NOT txid81077 [ORGN]” was used to remove artificial sequence hits.

### Identification of known and predicted pan-DENV HLA supertype binding sequences

Both literature search and query against the Immune Epitope Database [34] (www.immuneepitope.org) were performed to detect reported immunogenic, human T-cell determinants (both class I and II) of DENV that either fully or partially overlapped with the pan-DENV sequences. In addition, dedicated algorithms based on several prediction models were used to identify candidate putative HLA-binding sequences to multiple HLA class I and II supertype alleles within the pan-DENV sequences. Putative HLA supertypes class I-restricted peptides were identified by use of NetCTL [35], Multipred [36], ARB [37], and class II-restricted peptides by Multipred and TEPITOPE [38]. Further, the intra-type representation of the putative T-cell determinants was analyzed.

The NetCTL 1.2 algorithm (www.cbs.dtu.dk/services/NetCTL/) predicts peptides restricted by 12 HLA class I supertypes (A1, A2, A3, A24, A26, B7, B8, B27, B39, B44, B58 and B62). The algorithm integrates the predictions of HLA binding, proteasomal C-terminal cleavage and transport efficiency by the transporter associated with antigen processing (TAP) molecules. HLA binding and proteasomal cleavage predictions are performed by an artificial neural networks (ANN) method, while TAP transport efficiency is predicted using a weight matrix method. The parameters used for NetCTL prediction were: 0.15 weight on C terminal cleavage (default), 0.05 weight on TAP transport efficiency (default), and 0.5 threshold for HLA supertype binding, which was reported to be optimal (sensitivity (SN), 0.89 and specificity (SP), 0.94) in a large benchmark study containing more than 800 known class I T-cell determinants [35].

The TEPITOPE software (2000 beta version; courtesy of J. Hammer) utilizes quantitative matrix-based motifs, obtained from experimental scanning of the binding of P1-anchored designer peptides to soluble HLA-DR molecules in *in-vitro* competition assays, to predict peptides binding to 25 common HLA-DR alleles (DRB1*0101, *0102, *0301, *0401, *0402, *0404, *0405, *0410, *0421, *0701, *0801, *0802, *0804, *0806, *1101, *1104, *1106, *1107, *1305, *1307, *1311, *1321, *1501, *1502, and DRB5*0101) [38],[39]. The parameters for TEPITOPE predictions were: 5% quantitative threshold and putative determinants with a 10-fold inhibitory residue included. Nonamer peptides predicted to bind at least 10 out of the 25 HLA-DR alleles were selected as putative supertype-restricted determinants.

Multipred (research.i2r.a-star.edu.sg/multipred/) is a computational system for the prediction of peptides that bind to HLA class I supertypes A2 and A3 and class II HLA-DR supertype [36]. The HLA alleles selected to represent these supertypes by Multipred were as follows: A2 supertype, A*0201, *0202, *0203, *0204, *0205, *0206, *0207 and *0209; A3 supertype, A*0301, *0302, *1101, *1102, *3101, *3301 and *6801; DR supertype, DRB1*0101, *0301, *0401, *0701, *0801, *1101, *1301, and *1501. Hidden Markov model (HMM) and ANN methods are the predictive models of Multipred; both have been optimized and show similar performances [36]. The sum thresholds used for prediction of peptides restricted to the three HLA supertypes by ANN and HMM methods were: A2, 31.33 (ANN; SN = 0.80 and SP = 0.83) and 47.08 (HMM; SN = 0.80 and SP = 0.78); A3, 24.53 (ANN; SN = 0.90 and SP = 0.95) and 37.58 (HMM; SN = 0.80 and SP = 0.87); and DR, 23.42 (ANN; SN = 0.90 and SP = 0.92) and 51.08 (HMM; SN = 0.90 and SP = 1.00). Consensus predictions of the two methods were taken as final predictions for each HLA supertype.

The ARB matrix method (epitope.liai.org:8080/matrix/matrix_prediction.jsp) is based on a matrix of coefficients to predict IC50 values [37]. The HLA class I alleles predicted by ARB were grouped according to the current supertype classification [19],[40] and supertypes containing more than two alleles were selected, namely A2 (A*0201, *0202, *0203, *0206, and *6802), A3 (A*0301, *1101, *3101, *3301 and *6801), B7 (B*0702, A*3501, *5101, *5301, and *5401), and B44 supertypes (B*4001, *4002, *4402, *4403, and *4501). The prediction threshold value chosen for optimum sensitivity and specificity was IC50≤1000 nM and nonamer peptides predicted to bind 3 or more alleles of the supertype were considered as putative promiscuous HLA supertype-restricted determinants.

### ELISpot analysis of HLA-DR restricted determinants in pan-DENV sequences

All experiments were approved by the Johns Hopkins University Institutional Animal Care and Use Committee. Murine H-2 class II-deficient, HLA-DR2 [41], HLA-DR3 [42],[43], HLA-DR4 (referred to as DR4/IE) [44] and HLA-DR4/human CD4 (huCD4) [45],[46] Tg mice were used, bred and maintained in the Johns Hopkins University School of Medicine Animal Facility. Specific pathogen-free (SFP) colonies were maintained in a helicobacter-negative mice facility. The HLA-DR expression of the experimental transgenic mice was evaluated by flow cytometry.

Mice were immunized subcutaneously at the base of the tail, twice at two weeks interval, with pools of overlapping peptides covering the DENV-3 protein (15–17 aa, overlapping by 10–11 aa) (Schafer-N Inc., Copenhagen, Denmark; BEI Resources, Manassas, VA). Peptide pools (73–155 peptides per pool) contained 1 µg of each peptide and were emulsified (1∶1) in TiterMax adjuvant (TiterMax USA, Inc.). An aqueous preparation of TiterMax (1∶1) was used as a negative control. Two weeks after the second immunization, the mice were sacrificed and HLA-DR-restricted CD4 T cell reponses were assessed by *ex vivo* IFN-γ ELISpot assay using CD8-depleted splenocytes. Each target peptide was tested in duplicate. Spot-forming cell (SFC) counts were normalized to 10^6^ cells. The results were considered significant when the average SFC minus two standard deviations (SD) was greater than the average of the background plus two SD; and the average values were greater than 10 SFC per 10^6^ splenocytes. The initial screening assays were performed with peptide matrices [47], followed by assays with the relevant individual peptides (Nascimento *et al*., manuscript in preparation).

## Results

### Dengue virus type protein datasets

A total of 9,512 and 12,404 complete and partial DENV protein sequences were collected from the NCBI Entrez protein database of December 2005 and 2007, respectively, representing an increase of approximately 30% (2892 sequences) in the 24-months interval (Table 1). The total number of sequences (2007) varied from 4,011 for DENV-2 to 1,415 for DENV-4 and from 3,845 for E to 523 for NS4a proteins. Most of the individual protein sequences originated from DENV strains that were unique variants with respect to the entire polyprotein, but were identical to other strains with respect to individual proteins [48].

**Table 1:** Number and distribution of reported DENV protein sequences.

DENV protein^b^	No. of sequences^a^										
	DENV-1		DENV-2		DENV-3		DENV-4		*Total*		
	2005	2007	2005	2007	2005	2007	2005	2007	2005	2007	Increase
C	194	298	266	311	414	547	117	122	991	1278	287
prM	206	311	353	404	458	590	207	225	1224	1530	306
E	852	1051	1277	1518	716	910	338	366	3183	3845	662
NS1	410	565	640	752	201	308	142	159	1393	1784	391
NS2a	150	238	132	173	90	169	121	125	493	705	212
NS2b	136	224	130	163	104	183	40	44	410	614	204
NS3	98	186	145	178	216	297	30	34	489	695	206
NS4a	91	178	128	162	70	151	28	32	317	523	206
NS4b	89	176	129	163	70	150	109	113	397	602	205
NS5	92	179	151	187	181	267	191	195	615	828	213
*Total*	2318	3406	3351	4011	2520	3572	1323	1415	9512	12404	2892

a Collected from the NCBI Entrez protein database

b Manually processed after multiple sequence alignments and use of the known DENV cleavage sites

### Conserved pan-DENV sequences

**Figure 2:**
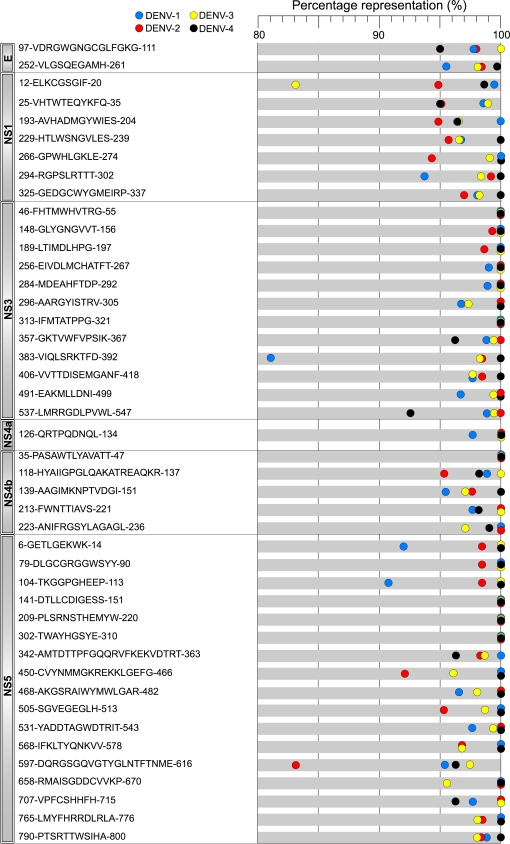
Pan-DENV sequences and their representations in the 4 DENV types. The 44 pan-DENV sequences of at least 9 amino acids that were found present in ≥80% of the recorded sequences of each DENV type are shown. The representation values are shown for the 2005 dataset; see Table S1 for values of both 2005 and 2007 datasets. Amino acid positions were numbered according to the sequence alignments of the 4 DENV types. The corresponding proteins are indicated on the left.

The consensus-sequence approach [20],[25] identified a total of 44 pan-DENV sequences of at least 9 amino acids that were present in ≥80% of all sequences of each DENV type for both 2005 and 2007 datasets (**Figure 2** **; Table S1**). Strikingly, 34 of the 44 (∼77%) were conserved in ≥95% of all reported DENV sequences. The size of the pan-DENV sequences ranged from 9 to 22 amino acids, with a combined size of 514 residues, corresponding approximately to 15% of the complete DENV polyprotein (∼3390 amino acids) (**Table 2**). The vast majority (42/44) of the pan-DENV sequences were localized in the NS proteins, with 17, 12, 7 and 5 sequences found in NS5, NS3, NS1 and NS4b, respectively, and 1 in the NS4a protein. Notably, the remaining two pan-DENV sequences were localized in the E protein. No region of at least 9 amino acids and conserved in ≥80% of the sequences of each DENV type was found in the C, prM, NS2a and NS2b proteins. The largest size of the combined pan-DENV sequences was in the NS5 protein, representing a total of 215 amino acid positions covering ∼24% of the protein, followed by NS3, NS1 and NS4b with 122, 74 and 69 amino acid positions covering ∼20, ∼21 and ∼28% of the corresponding proteins, respectively. The two pan-DENV sequences in the E protein had a combined size of only 25 amino acids, corresponding to ∼5% of the protein.

**Table 2:** Distribution and size of the pan-DENV sequences.

DENV protein	Size (aa)	Pan-DENV sequences^a^		
		No.	Size^b^	% of protein^c^
C	113–115	0	0	0
prM	166	0	0	0
E	493–495	2	25	5
NS1	352	7	74	21
NS2a	218	0	0	0
NS2b	130	0	0	0
NS3	618–619	12	122	20
NS4a	150	1	9	6
NS4b	245–249	5	69	28
NS5	900–904	17	215	24
*Total*	3387–3398	44	514	15

a Sequences of at least 9 amino acids that were represented in ≥80% of all DENV sequences of each type

b Combined amino acid size of all pan-DENV sequences in the protein

c Percentage of the combined pan-DENV sequence size over that of the corresponding protein size

In large-scale genomic analyses such as this study, biases may result from the collection of completely or partially overlapping redundant sequences, corresponding to identical or highly similar circulating DENV isolates sequenced by various dengue surveillance programs in different countries. Although to some extent this redundancy may be accepted as a reflection of the incidence of the corresponding DENV isolates in nature, we assessed its potential bias effect by repeating the analysis of conservation after discarding duplicate sequences from the datasets. The analysis of unique sequences identified all the pan-DENV sequences that were identified when including duplicates (**Figure 2**), except for NS1_12–20_, NS1_25–35_ and NS5_597–616_. Therefore, the presence of duplicates in the DENV datasets did not significantly affect the results. Although the removal of duplicates does not fully compensate for biases in the datasets, the removal of highly similar sequences, which may have been generated from relatively large sequencing efforts in single outbreaks, was deemed undesirable, since such arbitrary selection would introduce additional biases.

### Evolutionary diversity of DENV protein nonamer peptide sequences

**Figure 3:**
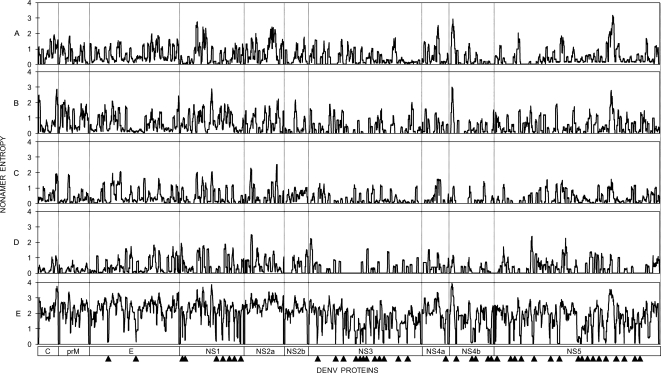
Shannon entropy of nonamer peptides within and across DENV types sequences. The entropy values were computed from the alignments of DENV sequences using the Antigenic Variability Analyzer software, as described in the *Methods*. Values were plotted for DENV-1 (A), DENV-2 (B), DENV-3 (C), DENV-4 (D), and all 4 DENV types (E) sequences (2005 dataset). Entropy values around protein cleavage sites are non significant, since the corresponding positions cannot be the center of a nonamer (*see* *Methods*). The triangles below indicate the locations of the pan-DENV sequences in the corresponding proteins.

The evolutionary diversity of each DENV type, and the 4 types combined, was studied by use of Shannon information entropy [27], modified to examine the variability of nonamer peptide sequences, as described in the *Methods*. The entropy of the proteome of the recorded viruses of each type showed numerous long regions of low entropy (≤1), reflecting the relatively high degree of intra-type sequence conservation, in particular in the NS3, NS4b and NS5 proteins (**Figure 3A–D** **)**. Overall, the average intra-type nonamer entropy values of the individual protein sequences of DENV-1, -2, -3 and -4 ranged from 0.2 for the DENV-4 NS4b to 1.0 for DENV-2 prM (**Figure S1**). Of note, however, were the marked differences in the relative degree of entropy of each protein between the 4 DENV types. For example, NS4b had the least diversity of the proteins of 3 types, but was replaced in DENV-2 by NS2b, which was the second most variable in DENV-3. The consequence of the differences in the sequences of each protein between the 4 types was a marked increase in the peptide entropy across the DENV 1-4 proteomes (**Figure 3E** **),** with average peptide entropy ranging from 1.6 for NS3 to 2.6 for NS2a **(Figure S1**), except for 44 sharply defined regions of low nonamer entropy (≤0.5) where the sequences were highly conserved in all DENVs (**Figure 3E** **)**, with no significant difference between the 2005 and 2007 datasets (**Table S2**). Majority of the pan-DENV sequences had entropy values of ≤0.3, corresponding to the intra-type representation of ≥90%. Thus, the congruent consensus- and entropy-based analyses of the DENV nonamer peptides revealed highly conserved and evolutionarily stable pan-DENV sequences distributed in several viral proteins, despite the marked viral diversity defining multiple DENV types, genotypes and variants [49].

### Representation of DENV variant nonamer peptide sequences

**Figure 4:**
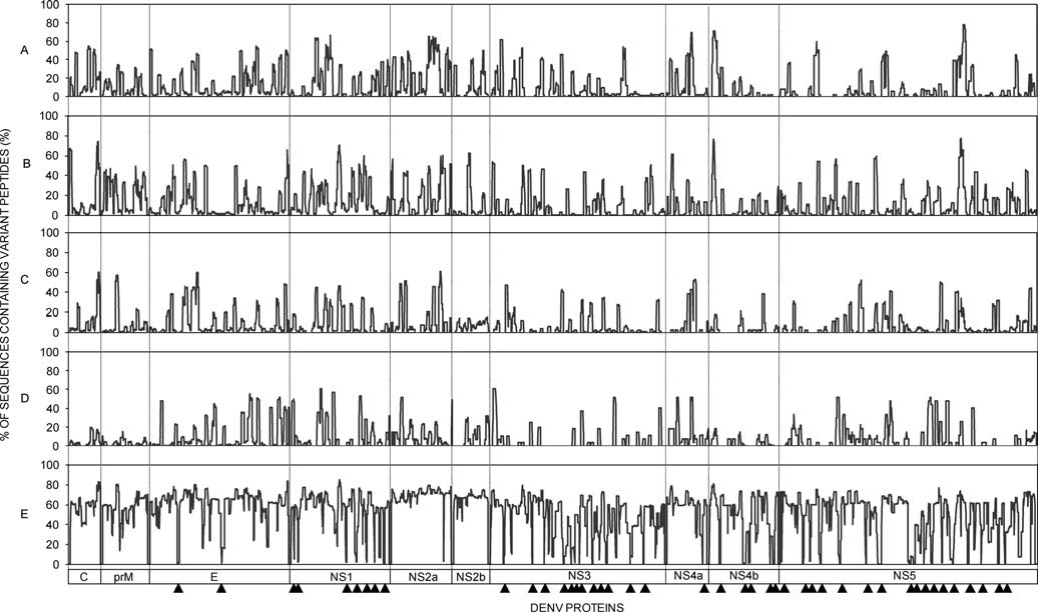
Percentage representations of variant nonamer peptides within and across DENV types sequences. The percentage of sequences that contained variant peptides at each nonamer position are shown for DENV-1 (A), DENV-2 (B), DENV-3 (C), DENV-4 (D), and all 4 DENV types (E) (2005 dataset). Values around protein cleavage sites are non significant (see Figure 3). The triangles below indicate the locations of the pan-DENV sequences in the corresponding proteins.

The combined representation of variant peptides that differed by at least one amino acid from the predominant peptide was also analyzed at each nonamer position. Examples of this analysis for DENV-3 proteins are shown in **Table 3**. Nonamers that lack entropy (zero entropy) have one sequence in all of the recorded virus isolates, and therefore have no variants. Positions with high entropy can contain many different variant peptides, each at lesser (or equal) frequency than the predominant peptide. The combined representation of variant peptides at each nonamer position across the proteome of each individual DENV type was generally low, representing less than 10% of the corresponding sequences, except for some positions where it was more than 50% (**Figure 4A–D**). Notably, the nonamer position with the highest combined variant representation for each DENV type was found in the nonstructural proteins and not the structural ones, with representation values ranging from ∼61 to ∼78% (DENV-1 NS5, DENV-2 NS5, DENV-3 NS2a, and DENV-4 NS1 and NS3 proteins). When representations of variants across all DENVs were calculated, the majority of all nonamer sites contained variants that together represented ∼60–85% of the total DENV sequences at that site (the highest representation of ∼85% was in the NS1 protein) (**Figure 4E**). This was in striking contrast to the 0 to ∼5% combined representation of variants at each nonamer position in the pan-DENV sequences, with no significant difference between the 2005 and 2007 datasets (**Table S2**). The majority of all nonamer sites in the pan-DENV sequences lacked variant or contained variants that together represented <1% of all recorded DENVs. These data further illustrate the extremely high genetic stability of the 44 pan-DENV sequences, among all recorded DENV sequences and demonstrate that irrespective of the high variability between the sequences of the 4 DENV types, the representation of variants in the pan-DENV sequences was almost negligible.

**Table 3:** Examples of the distribution of variant nonamer peptides in DENV-3.

DENV-3 protein	Nonamer position	No. of sequences	Nonamer peptides^a^	Representation of peptides	Combined % representation of variants^b^	Nonamer entropy^c^
E	14	479	DFVEGLSGA	479 (100%)	0	0
NS2a	176	64	LAGISLLPV	25 (39%)	61	2.4
			LAGVSLLPV	11 (17%)		
			LAGVSLLPL	9 (14%)		
			LAVISLLPV	9 (14%)		
			LAGISLLPL	6 (9%)		
			LAGISLFPV	2 (3%)		
			LAGISLMPV	2 (3%)		
NS4a	86	68	SIGLICVVA	39 (57%)	43	1.5
			SIGLICVIA	19 (28%)		
			SIGLICVIV	8 (13%)		
			SIGLICVAA	2 (3%)		

a The predominant peptide is underlined

b Variants include all the peptides at the position, except the predominant

c Entropy value of all the peptides at the position (predominant peptide included)

### Functional and structural correlates of the pan-DENV sequences

Highly conserved protein sequences are likely to represent critical sites and domains [50]. A search of the literature and the Prosite and Pfam databases [30],[32] revealed that 27 of the 44 pan-DENV sequences were associated with biological activities (**Table S3**); the functional significance of the remaining 17 pan-DENV sequences was not known. The two pan-DENV sequences in the E protein corresponded to the fusion peptide (positions 98 to 110) and dimerisation domain [51],[52]. In NS3, one pan-DENV sequence corresponded to the peptidase family S7 (*Flavivirus* serine protease) domain and comprised the His-51 catalytic residue [53], 3 sequences corresponded to known/putative *Flavivirus* Asp-Glu-Ala-Asp/His (DEAD/H) domain associated with ATP-dependent helicase activity [54], and two sequences were predicted to be required for cell attachment and targeting signal for microbodies. In NS5, one pan-DENV sequence corresponded to the conserved methyltransferase (MTase) *S*-adenosyl-L-methionine binding motif I (positons 77–86) involved in viral RNA capping [55], and two sequences corresponded to RNA dependent RNA polymerase (RdRp) domain [56]. Furthermore, 6 of the 27 pan-DENV sequences were predicted to exhibit post-translational modification(s), including N-glycosylation, protein kinase C and casein kinase II phosphorylation, N-myristoylation and/or amidation (**Table S3**).

It is generally recognized that amino acids buried inside proteins are subject to greater interactions and packing constraints [57] than those exposed on the outer surface. Although none of the DENV protein structures in the protein data bank (PDB) [33] was full-length, 19 of the 44 pan-DENV sequences could be mapped on the available crystallographic models of the E ectodomain (Accession No. 1OAN; 394 out of 493–495 residues), NS3 (1BEF and 2BMF, 181 and 451 out of 618–619 residues, respectively) and NS5 fragments (1R6A, 295 out of 900–904 residues). Eleven of the 19 pan-DENV sequences were buried, 2 partially exposed and 6 exposed at the surface of the corresponding structures (**Figure S2**). However, these results should be considered preliminary until full-length 3-D structures are available.

### Distribution of pan-DENV sequences in nature

**Figure 5:**
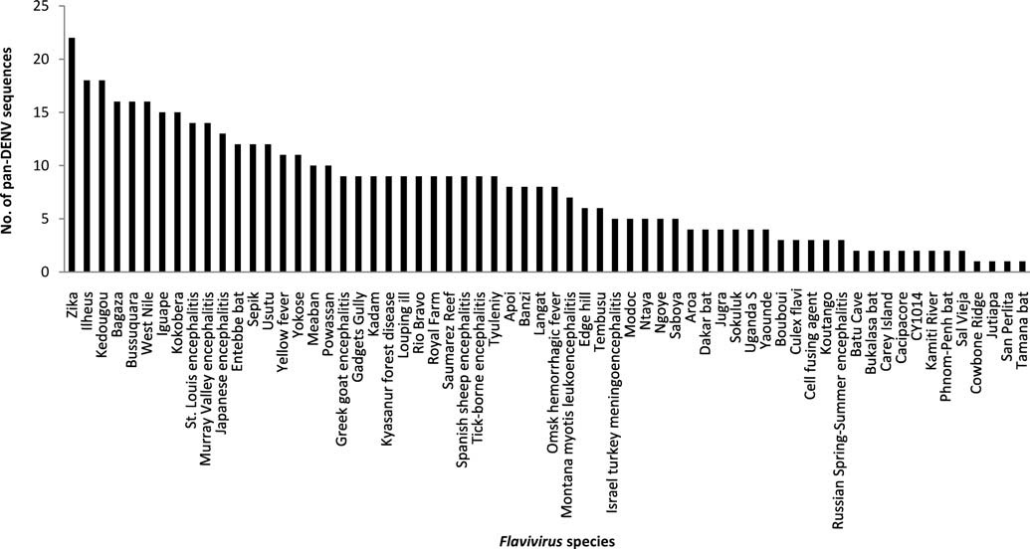
Number of pan-DENV sequences conserved in the different *Flaviviruses.*

**Figure 6:**
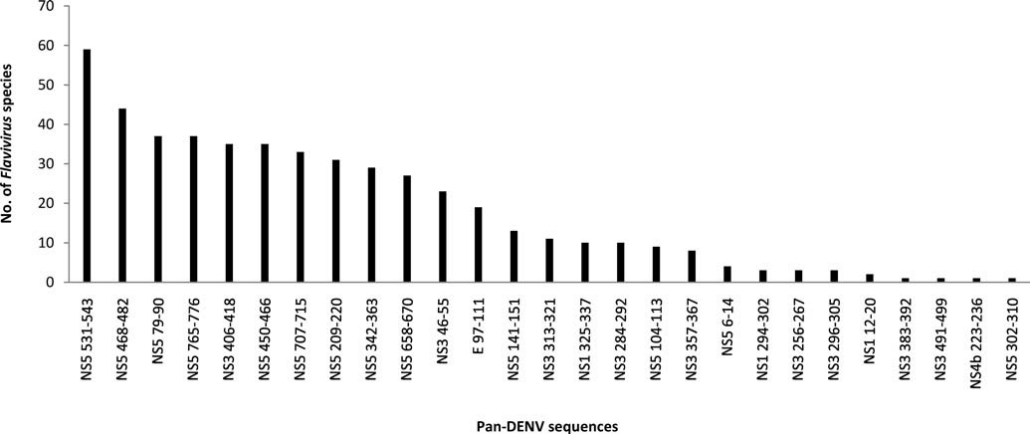
Number of *Flaviviruses* shared by the pan-DENV sequences.

Twenty-seven (27) of the 44 pan-DENV sequences overlapped at least 9 amino acid sequences of as many as 64 other viruses of the family *Flaviviridae,* genus *Flavivirus* (**Figure 5**). *Zika virus* shared 22 of the 27 sequences; *Ilheus* and *Kedougou* viruses, 18; and representatives of some of the significant human pathogens, *West Nile*, *St. Louis encephalitis*, *Japanese encephalitis*, *Yellow fever* and *Tick-borne encephalitis* viruses, shared from 16 to 9 pan-DENV sequences. Thirteen (13) of the 27 sequences represented NS5, of which 9 were present in at least 27 *Flavivirus* species; 9 represented NS3, of which two were found in 35 and 23 species; one E sequence was found in 19 species; and the remaining were in NS1 and NS4b (**Figure 6** **; Table S4**). Five (5) of the 27 were associated with known biological activities (NS5_79–90_ MTase, NS5_658–670_ RdRp, NS3_46–55_ peptidase S7, NS3_284–292_ DEAD/H and E_97–111_ dimerisation/fusion domains). Interestingly, two sequences, NS3_406–418_ and NS5_597–616_, overlapped 9 amino acid sequences of the cell fusing agent virus polyprotein-like protein from the mosquito *Aedes albopictus* [58], and the phage-related tail fibre protein-like protein from the bacteria *Chromohalobacter salexigens DSM 3043*, respectively.

The representation of many of the pan-DENV sequences was high among known sequences of several of the highly studied *Flaviviruses* (**Table S4**): *St. Louis encephalitis*, *West Nile*, *Japanese encephalitis*, *Murray Valley encephalitis*, *Usutu*, *Kokobera*, *Ilheus*, *Tick-borne encephalitis*, *Langat*, *Omsk hemorrhagic fever*, *Louping ill*, *Powassan*, *Kyasanur forest disease and Yellow fever* viruses. Protein sequence data for the rest of the *Flaviviruses* that shared pan-DENV sequences was limited (<10 sequences) in the public database. Seven of the 27 pan-DENV sequences, NS1_12–20_, NS3_256–267_, NS3_383–392_, NS3_491–499_, NS4b_223–236_, NS5_6–14_ and NS5_302–310_, were present in a few species with less than 10 reported total sequences (**Table S4**).

### Known and predicted HLA supertype-restricted, pan-DENV T-cell determinants

**Figure 7:**
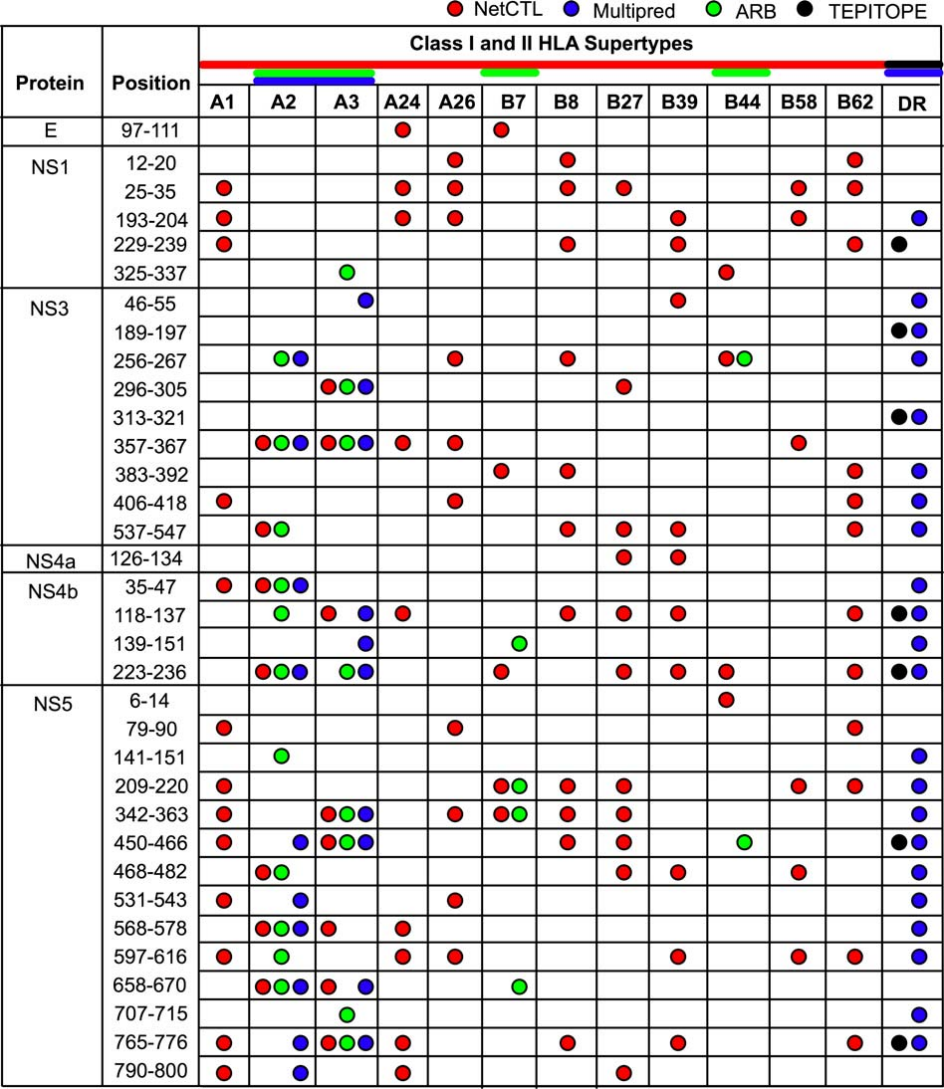
Candidate putative HLA supertype-restricted, pan-DENV T-cell determinants predicted by computational algorithms. Amino acid positions of the pan-DENV sequences are numbered according to the sequence alignments of the 4 DENV types; the corresponding DENV proteins are indicated on the left. Predicted HLA-restricted T-cell determinants were identified using NetCTL, Multipred, ARB and TEPITOPE algorithms (see Methods).

Literature survey and database search revealed that 10 of the pan-DENV sequences (9 in NS3, one in E) overlapped at least 9 amino acids of 15 previously reported DENV T-cell determinants immunogenic in human, with their HLA restriction, when known, showed both class II (DR*15, DPw2) and class I (A*11) specificities (**Table 4**). Further evaluation of the immune-relevance of the pan-DENV sequences included a search for candidate putative promiscuous HLA supertype-restricted T-cell determinants within these regions by use of several computational algorithms: NetCTL [35], Multipred [36], ARB [37] and TEPITOPE [38]. Overall, 34 of 44 (∼77%) pan-DENV sequences (**Figure 7**), identified in the NS5, NS3, NS1, E and NS4a proteins were predicted to contain 100 supertype-restricted binding nonamers (**Table S5**). The majority (88/100) of the predicted promiscuous HLA-binding nonamers were present in ≥95% of the sequences of each DENV type (**Table S6**). Thirty-one (∼91%) of the 34 putative supertype pan-DENV sequences contained HLA-binding nonamers for multiple HLA supertypes. Clusters (hotspots) of two or more overlapping HLA-binder nonamer core peptides were present in 27 (∼79%) of the 34 putative supertype pan-DENV sequences. About half (14/27) of these clusters contained three or more nonamer binders overlapping by 8 amino acids, covering most or the entire corresponding conserved region.

**Table 4:** Reported human T-cell determinants in the pan-DENV sequences.

DENV protein	Pan-DENV sequence^a^	Immunogenic T-cell determinants^b^			
		Sequence^c^	T subset	HLA Ag	Reference(s)
E	_252_VLGSQEGAMH_261_	KKQDVVVLGSQEGAM	**-**	*-*	[76]
NS3	_46_FHTMWHVTRG_55_	TFHTMWHVTRGAVLM	CD4	*-*	[76]
	_148_GLYGNGVVT_156_	KVVGLYGNGVVTRSG	CD4	DR*15	[76]
	_189_LTIMDLHPG_197_	KRLTIMDLHPGAGKT	CD4	**-**	[72]
		RKLTIMDLHPGSGKT	CD4	**-**	[72]
		RKLTIMDLHPGAGKT	CD4	**-**	[72]
		RNLTIMDLHPGSGKT	CD4	**-**	[72]
	_256_EIVDLMCHATFT_267_	EHTGREIVDLMCHAT	CD4	**-**	[76]
		EIVDLMCHATFTMRL	CD4	**-**	[76]
		EIVDLMCHAT	CD4	DPw2	[77],[78]
	_284_MDEAHFTDP_292_	LIIMDEAHFTDPASI	**-**	*-*	[76]
	_313_IFMTATPPG_321_	AGIFMTATPPGSRDP	**-**	**-**	[76]
	_357_GKTVWFVPSIK_367_	TVWFVPSIK	CD8	A*11	[16]
	_383_VIQLSRKTFD_392_	KKVIQLSRKTFDSEY	**-**	*-*	[76]
	_406_VVTTDISEMGANF_418_	NDWDFVVTTDISEMG	**-**	*-*	[76]

a Amino acid positions numbered according to the sequence alignments of the 4 DENV types

b Dashes, not determined

c Sequences present in the pan-DENV sequences are underlined

### Immunogenicity of HLA-DR-restricted pan-DENV sequences in HLA Tg mice

The immunogenicity of the pan-DENV sequences was also analyzed by assay of peptide-specific HLA-restricted T-cell responses in murine H-2 class II-deficient, HLA-DR Tg mice expressing 3 prototypic HLA-DR alleles, corresponding to the divergent subgroups HLA-DR2 (DRB1*1501), HLA-DR3 (DRB1*0301), and HLA-DR4 (DRB1*0401). Mice were immunized with pools of overlapping peptides covering the sequences of the E, NS1, NS3, and NS5 proteins of DENV-3, and HLA-DR-restricted CD4 T-cell responses were assessed by IFN-γ ELISpot assays using CD8-depleted splenocytes. Thirty peptides eliciting positive T-cell responses in the HLA Tg mice contained 9 or more consecutive amino acids of 22 pan-DENV sequences, that were localized in the NS5 (11), NS3 (6), NS1 (4), and E proteins (one) (**Table 5**). Overall, 9, 10 and 18 peptides elicited positive responses in HLA-DR2, -DR3, and/or -DR4 Tg mice, respectively; 20 corresponded to sequences of NS5, 10 of NS3, 6 of NS1, and one of E. Furthermore, at least 7 of the pan-DENV sequences, all localized in the NS5 and NS1 proteins, contained promiscuous T-cell determinants for multiple HLA-DR alleles (**Table 5**). These data, together with those previously reported **(** **Table 4** **),** showed that a minimum of 26 of the 44 pan-DENV sequences, distributed predominantly in the NS5 and NS3 proteins, and to a lesser extent in NS1 and E, contained numerous HLA-restricted class II and/or class I determinants demonstrated by assays of T-cell responses *in vivo*.

**Table 5:** Immunogenicity of the pan-DENV sequences in HLA-DR transgenic mice.

DENV protein	Pan-DENV sequence^b^	Ag-specific CD4 T-cell responses^a^			
		Peptide sequences (DENV-3)^c^	IFN-γ-SFC/10^6^splenocytes±SD^d^		
			DR2	DR3	DR4
E	_252_VLGSQEGAMH_261_	PEVVVLGSQEGAMHT	-	-	88±34
NS1	_193_AVHADMGYWIES_204_	AVHADMGYWIESQKN	-	17±1	-
	_229_HTLWSNGVLES_239_	WPKSHTLWSNGVLES	-	129±3*	-
		HTLWSNGVLESDMII	-	131±103	37±3
	_266_GPWHLGKLE_274_	HTQTAGPWHLGKLE	-	333±6	-
	_294_RGPSLRTTT_302_	TRGPSLRTTTVSGKL	-	-	11±4
NS3	_189_LTIMDLHPG_197_	KKRNLTIMDLHPGSG	-	-	50±16
	_296_AARGYISTRV_305_	ASIAARGYISTRVGM	40±14	-	-
		ARGYISTRVGMGEAA	9±4	-	-
	_313_IFMTATPPG_321_	EAAAIFMTATPPGTA	-	-	474±116
		IFMTATPPGTADAFP	-	-	323±287
	_357_GKTVWFVPSIK_367_	TDFAGKTVWFVPSIK	48±15	-	-
		GKTVWFVPSIKAGND	396±14	-	-
	_383_VIQLSRKTFD_392_	KKVIQLSRKTFDTEY	-	21±3	-
	_406_VVTTDISEMGANF_418_	FVVTTDISEMGANFK	-	-	408±104
		TDISEMGANFKADRV	-	152±33	-
NS5	_302_TWAYHGSYE_310_	DENPYKTWAYHGSYEVK	126±10*	-	14±5
		TWAYHGSYEVKATGSA	161±20*	-	63±17
	_342_AMTDTTPFGQQRVFKEKVDTRT_363_	MVTQMAMTDTTPFGQQR	-	-	28±0*
	_450_CVYNMMGKREKKLGEFG_466_	GSCVYNMMGKREKKLGE	-	-	13±2
	_505_SGVEGEGLH_513_	NSYSGVEGEGLHKLGYI	-	-	184±15
	_531_YADDTAGWDTRIT_543_	KIPGGAMYADDTAGWDT	-	-	46±3
	_568_IFKLTYQNKVV_578_	ANAIFKLTYQNKVVKVQ	577±384	-	24±9*
	_597_DQRGSGQVGTYGLNTFTNME_616_	VMDIISRKDQRGSGQVG	-	88±1	-
	_658_RMAISGDDCVVKP_670_	VERLKRMAISGDDCVVK	-	159±24	16±6
		MAISGDDCVVKPIDDRF	-	249±39	-
	_707_VPFCSHHFH_715_	DWQQVPFCSHHFHELIM	32±8*	34±11	-
	_765_LMYFHRRDLRLA_776_	MYFHRRDLRLASNAI	75±16*	-	33±9
	_790_PTSRTTWSIHA_800_	VHWVPTSRTTWSIHAHH	-	-	83±1
		SRTTWSIHAHHQWMTTE	-	-	122±46

a Assessed by IFN-γ ELISpot assay in HLA-DR2 (DRB1^*^1501), HLA-DR3 (DRB1^*^0301) and HLA-DR4 (DRB1^*^0401) Tg mice immunized with DENV-3 peptides (see Methods)

b Amino acid positions numbered according to the sequence alignments of the 4 DENV types

c Sequences present in the pan-DENV sequences are underlined

d SFC, spot-forming cells; SD, standard deviation. Representative results from at least two immunized Tg mice are shown, except when indicated by an asterisk

## Discussion

In this study, we identified and characterized pan-DENV sequences that were highly conserved in all recorded DENV isolates. The large number of sequences analyzed (12,404 as of December 2007), and their wide distribution in terms of geography and time (1945–2007) (data not shown), offered information for a broad survey of DENV protein diversity in nature. The 44 pan-DENV protein sequences of at least 9 aa, covering 514 aa or about 15% of the complete DENV polyprotein of ∼3390 aa, were conserved in at least 80% of all recorded DENV sequences, and 34 of the 44 (∼77%) were conserved in ≥95% of DENV sequences. All the 44 were in the non-structural proteins except for the two E sequences. These conserved sequences have shown remarkable stability over the entire history of DENV sequences deposited in the NCBI Entrez protein database, as illustrated by their low peptide entropy values and variant frequencies. In addition, 27 of the pan-DENV sequences were conserved in 64 other *Flaviviruses,* as further evidence of prolonged evolutionary stability within this genus, as previously discussed [59]–[61]. Two are also present in the proteomes of the *Aedes albopictus* mosquito and the bacteria *Chromohalobacter salexigens*, possibly in keeping with recent reports of the genetic recombination between phyla [58]. It is likely that these pan-DENV sequences have been under selection pressure to fulfill critical biological and/or structural properties, some of which have been identified for the E (fusion peptide, dimerization domain), NS3 (peptidase S7, DEAD/H domains) and NS5 proteins (MTPase, RdRp domains) [51]–[56]. Hence, these conserved sequences are unlikely to significantly diverge in newly emerging DENV isolates in the future, and represent attractive targets for the development of specific anti-viral compounds and vaccine candidates.

There also is evidence that many of the conserved sequences are immunologically relevant. A majority (26/44) contained at least 9 amino acids overlapping with a total of 45 peptides that have been reported to be immunogenic in humans and/or HLA-DR Tg mice. In addition, putative T-cell determinants for 12 major HLA class I supertypes and for class II DR supertype, with broad application to the immune responses of human population worldwide, were predicted by computational analysis. Some of the putative T-cell determinants were predicted to be promiscuous to multiple HLA supertypes, in addition to multiple alleles of a given HLA supertype. Such a degree of promiscuity has previously been observed for DENV [62] and HIV peptides [63], among others. The existence of conserved T-cell determinants specific for multiple HLA supertypes further supports their evaluation as vaccine targets, since they would provide broader population coverage [63]. Many of the predicted HLA binding nonamers were localized in clusters, as we have also observed in HLA Tg mice immunized with WNV proteins and DNA encoding the SARS coronavirus N protein [64], and has been reported in studies of *human immunodeficiency virus* (HIV) type 1 proteins [65]–[68], the outer membrane protein of *Chlamydia trachomatis* [69], and other antigens [64].

The significant sequence variations between the proteins of the 4 DENV types represent a cardinal issue for the development of a tetravalent DENV vaccine that provides robust protection against each DENV type. Subtle amino acid substitutions within T-cell determinants restricted by a given HLA allomorph, such as in the event of sequential heterologous infections, or between a vaccine formulation and a subsequent natural infection [7], can dramatically alter the phenotype of the specific T cells, resulting in a wide range of effects from agonism to antagonism [9], [12]–[15]. Because of the extent of intra-type (1 to 21%) and inter-type (14 to 67%) amino acid variability among DENV isolates [48], many nonamer T-cell determinants contain single or multiple amino acid difference(s). When the 4 DENV types were analyzed together, a majority of the nonamer positions across the full proteome exhibited variants that together were present in ∼60 to ∼85% of all sequences. The frequencies of variant peptides across the 4 DENV types suggest that vaccine strategies incorporating whole DENV immunogens, such as inactivated and recombinant subunit vaccines, live attenuated viruses, or chimeric viruses expressing structural DENV genes, are likely to elicit T-cell responses to altered peptide ligands. This phenomenon is also likely to occur in individuals exposed to several *Flaviviruses*, such as DENV, JEV and YFV that are co-circulating in regions of Asia, India or South America, or following vaccination [70].

While the immune correlates of DENV protection remain poorly documented, there is evidence that both neutralizing antibody and specific T-cell responses are required [7],[71]. The incorporation of defined HLA-restricted T-cell determinants within DENV vaccine candidates might improve vaccine efficiency by increasing T-cell help to sustain a robust, long-lived immunity, and possibly through direct cytostatic and cytotoxic effects on infected cells. For tetravalent formulations, it may be relevant to focus primarily on sequences that are conserved in all 4 DENV types and to avoid the regions of T-cell immunity that are highly variable, unless they are strictly type-specific [17],[72]. The two pan-DENV E sequences (positions 97–111 and 252–261) and the exposed domain III of the E antigen (positions 300–400) [73],[74], are also candidate sequences for neutralizing antibody responses. An additional criterion for the selection of T-cell targets is the need for determinants with broad HLA representation, as it has been emphasized in the recognition of HLA supertypes [18]–[20]. Further investigations are needed to validate the immunogenicity of the candidate T-cell determinants in human subjects, and to identify sequences associated with deleterious T-cell responses.

The global approach described herein provides a framework and methodology for large-scale and systematic analysis of conserved sequences of other pathogens, in particular for rapidly evolving viruses such as influenza A virus [75] and HIV [63]. These studies will offer insights into their diversity and evolutionary history, together with providing critical data for rational vaccine development, structure-based design of candidate inhibitory compounds, and improvement of the current diagnostic methods.

## Supporting Information

Figure S1 Average nonamer peptide entropy for each protein of each DENV type and all the four types combined. The values are shown for the 2005 dataset.
(0.70 MB TIF)

Figure S2 Molecular location of 19 pan-DENV sequences (in red) on the protein's 3-D structure. These sequences were mapped on the available crystallographic models of the E ectodomain (PDB Accession No. 1OAN; 394 out of 493-495 residues), NS3 (1BEF and 2BMF, 181 and 451 out of 618-619 residues, respectively) and NS5 fragments (1R6A, 295 out of 900-904 residues). The major portions of eleven of the 19 pan-DENV sequences were buried (NS3-_148_GLYGNGVVT_156_, _256_EIVDLMCHATFT_267_, _284_MDEAHFTDP_292_, _296_AARGYISTRV_305_, _313_IFMTATPPG_321_, _357_GKTVWFVPSIK_367_, _406_VVTTDISEMGANF_418_, and _491_EAKMLLDNI_499_; NS5-_79_DLGCGRGGWSYY_90_, _141_DTLLCDIGESS_151_ and _209_PLSRNSTHEMYW_220_), 2 were partially buried/exposed (NS3-_46_FHTMWHVTRG_55_ and _537_LMRRGDLPVWL_547_) and the remaining 6 were exposed (E-_97_VDRGWGNGCGLFGKG_111_ and _252_VLGSQEGAMH_261_; NS3-_189_LTIMDLHPG_197_ and _383_VIQLSRKTFD_392_; NS5-_6_GETLGEKWK_14_ and _104_TKGGPGHEEP_113_) at the surface of the corresponding structures.
(9.65 MB DOC)

Table S1 The intra-type percentage representation of pan-DENV sequences.
(0.10 MB DOC)

Table S2 Pan-DENV sequences, entropy and representation of variants.
(0.08 MB DOC)

Table S3 Functional and structural properties of pan-DENV sequences.
(0.06 MB DOC)

Table S4 Distribution of pan-DENV sequences in nature.
(0.12 MB DOC)

Table S5 Candidate putative HLA supertype-restricted binding nonamer peptides in pan-DENV sequences, predicted by immunoinformatic algorithms.
(0.20 MB DOC)

Table S6 Intra-type representation of candidate putative HLA supertype-restricted nonamer peptides predicted by immunoinformatics algorithms.
(0.22 MB DOC)

Dataset S1 GI numbers.
(0.86 MB XLS)

Alternative Language Abstract S1 Translation of the abstract into Chinese by Guang Lan Zhang.
(0.06 MB PDF)
