# Supplementary figures and images for: Conservation and Variability of Dengue Virus Proteins: Implications for Vaccine Design
Source: PLoS Negl Trop Dis. 2008 Aug 13;2(8):e272. doi: 10.1371/journal.pntd.0000272 (PMC2491585; doi:10.1371/journal.pntd.0000272)

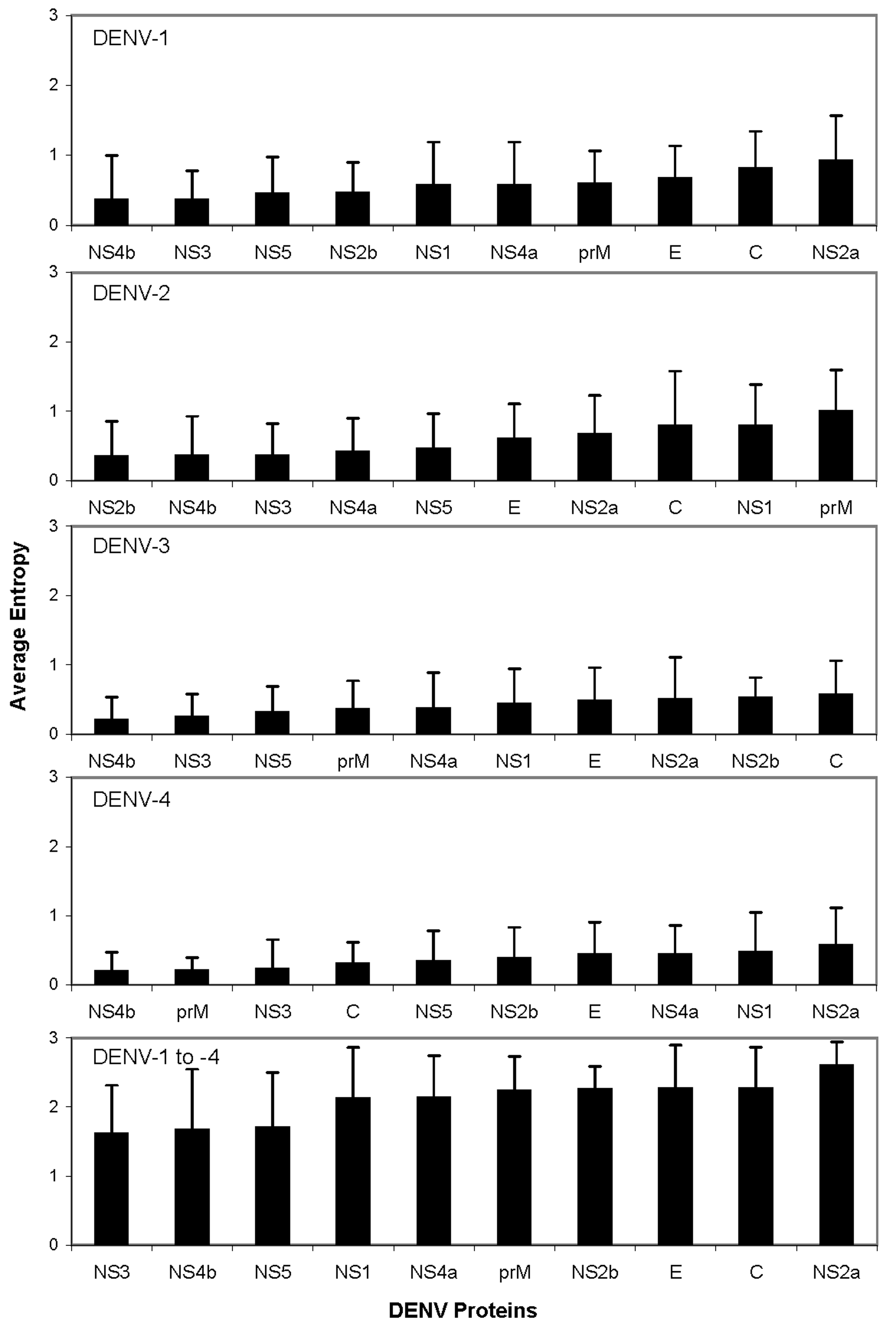

Supplement: Figure S1 — Average nonamer peptide entropy for each protein of each DENV type and all the four types combined. The values are shown for the 2005 dataset. (0.70 MB TIF) [file pntd.0000272.s001.tif]
