# Supplementary material for: Conservation and Variability of Dengue Virus Proteins: Implications for Vaccine Design
Source: PLoS Negl Trop Dis. 2008 Aug 13;2(8):e272. doi: 10.1371/journal.pntd.0000272 (PMC2491585; doi:10.1371/journal.pntd.0000272)
Supplement: Figure S2 — Molecular location of 19 pan-DENV sequences (in red) on the protein's 3-D structure. These sequences were mapped on the available crystallographic models of the E ectodomain (PDB Accession No. 1OAN; 394 out of 493-495 residues), NS3 (1BEF and 2BMF, 181 and 451 out of 618-619 residues, respectively) and NS5 fragments (1R6A, 295 out of 900-904 residues). The major portions of eleven of the 19 pan-DENV sequences were buried (NS3-148GLYGNGVVT156, 256EIVDLMCHATFT267, 284MDEAHFTDP292, 296AARGYISTRV305, 313IFMTATPPG321, 357GKTVWFVPSIK367, 406VVTTDISEMGANF418, and 491EAKMLLDNI499; NS5-79DLGCGRGGWSYY90, 141DTLLCDIGESS151 and 209PLSRNSTHEMYW220), 2 were partially buried/exposed (NS3-46FHTMWHVTRG55 and 537LMRRGDLPVWL547) and the remaining 6 were exposed (E-97VDRGWGNGCGLFGKG111 and 252VLGSQEGAMH261; NS3-189LTIMDLHPG197 and 383VIQLSRKTFD392; NS5-6GETLGEKWK14 and 104TKGGPGHEEP113) at the surface of the corresponding structures. (9.65 MB DOC) [file pntd.0000272.s002.doc]

| 1) E(_97_VDRGWGNGCGLFGKG_111_)  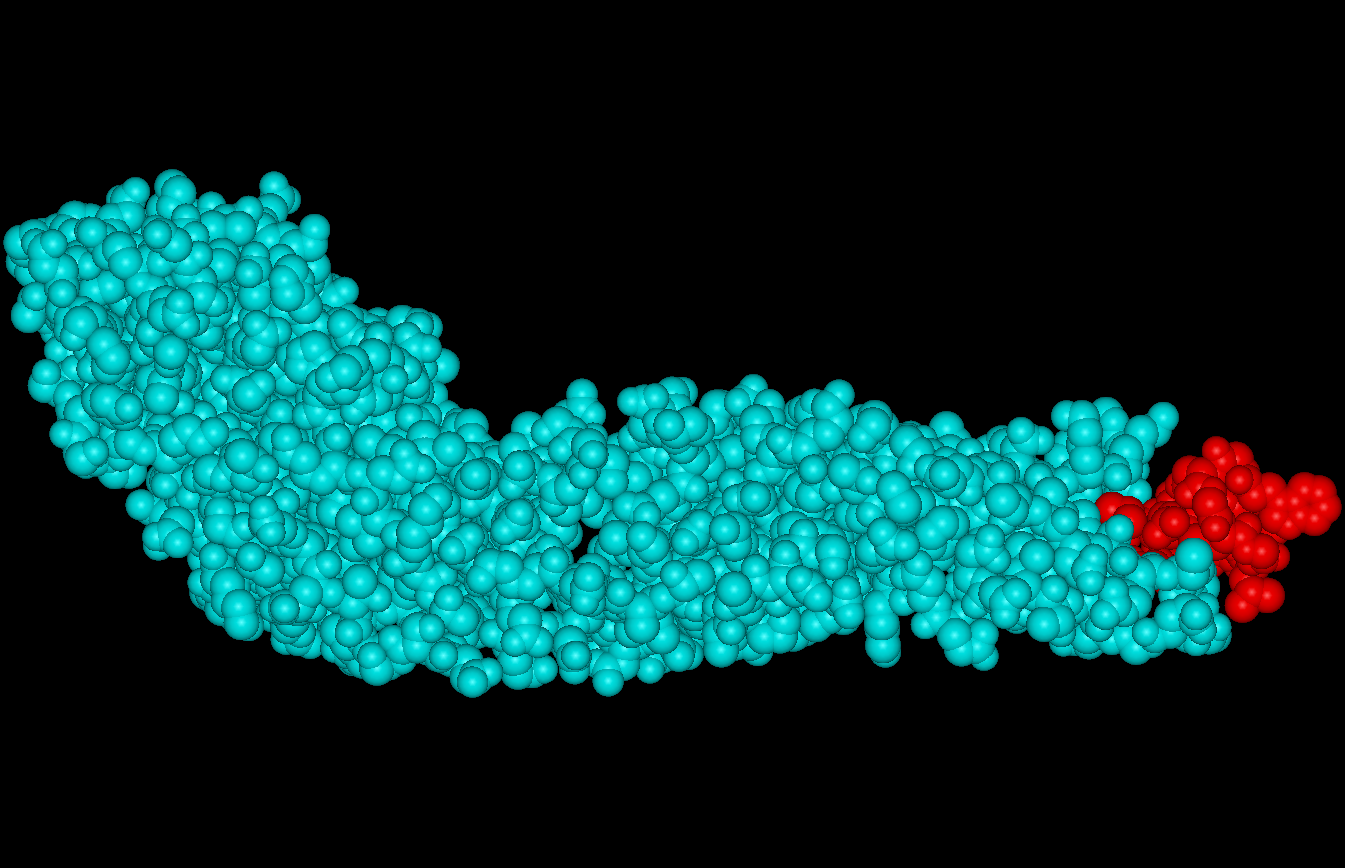 | 2) E(_252_VLGSQEGAMH_261_)  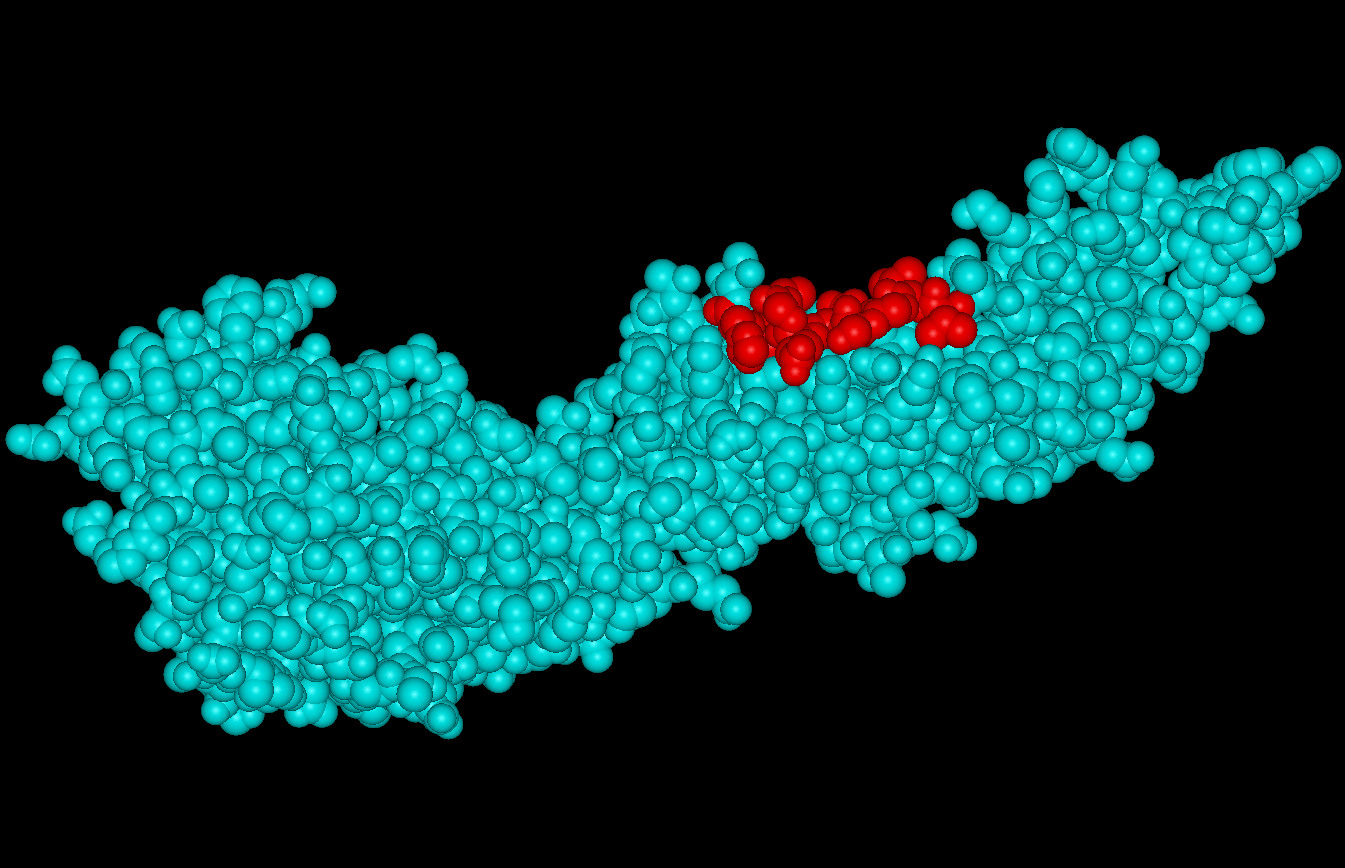 |
| --- | --- |
| 3) NS3(_46_FHTMWHVTRG_55_)  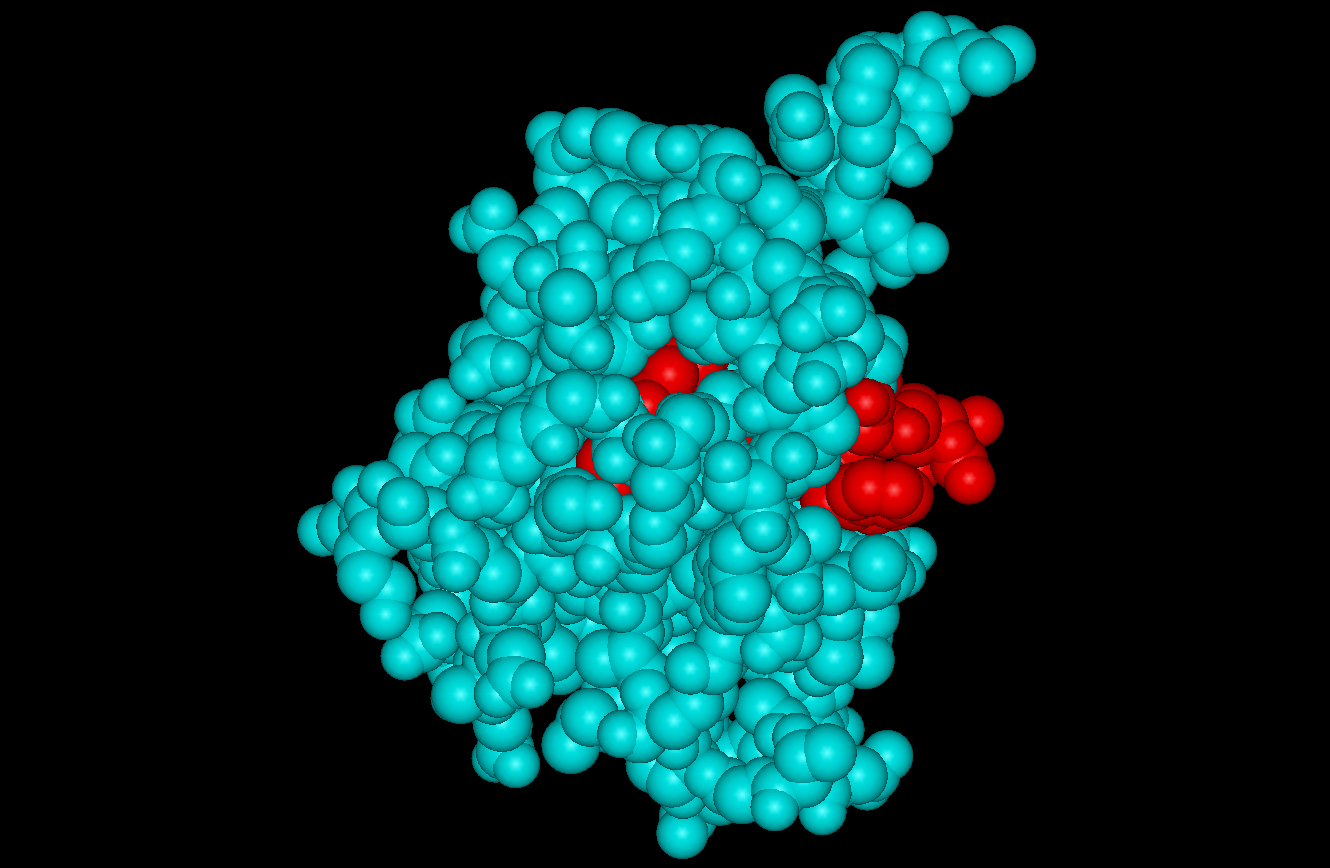 | 4) NS3(_148_GLYGNGVVT_156_)  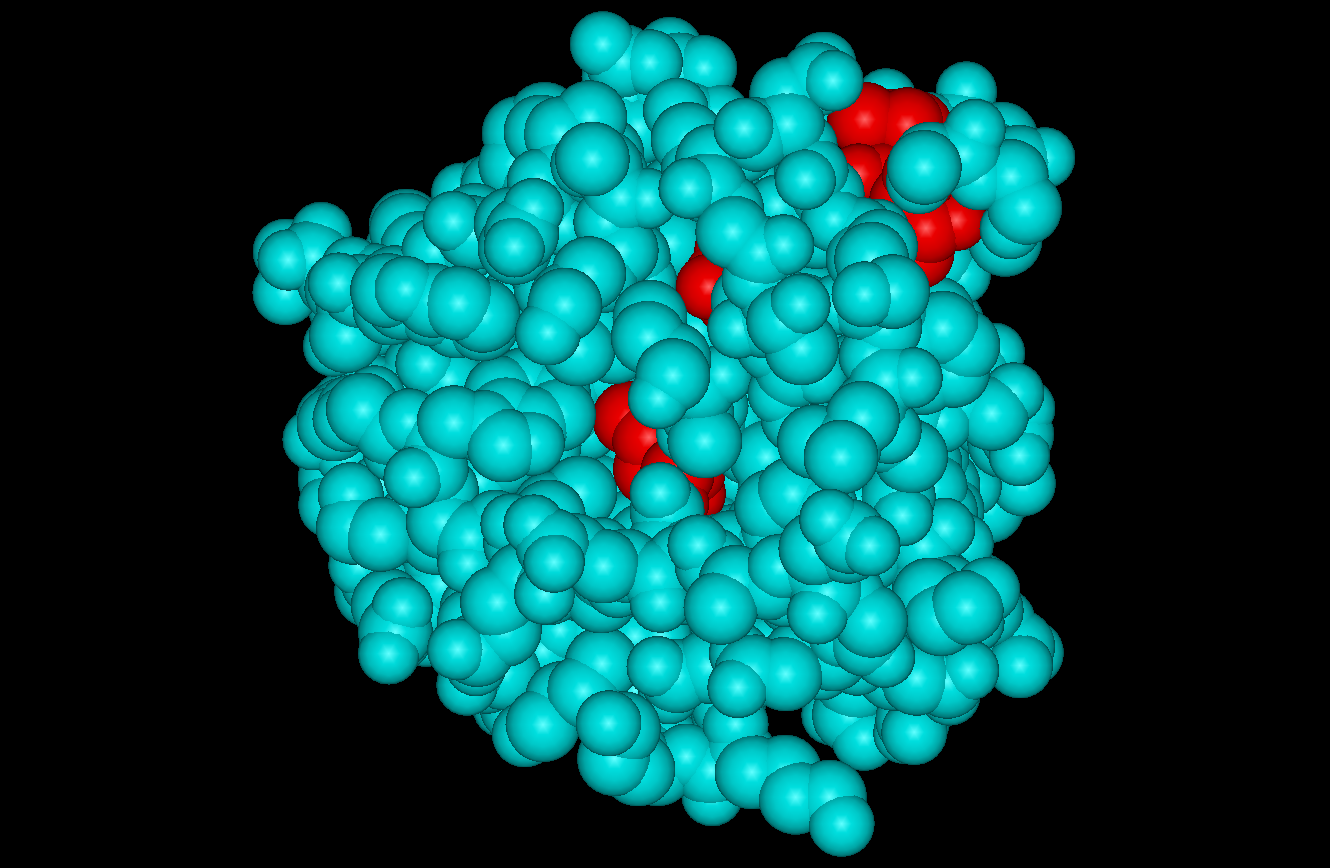 |
| 5) NS3(_189_LTIMDLHPG_197_)  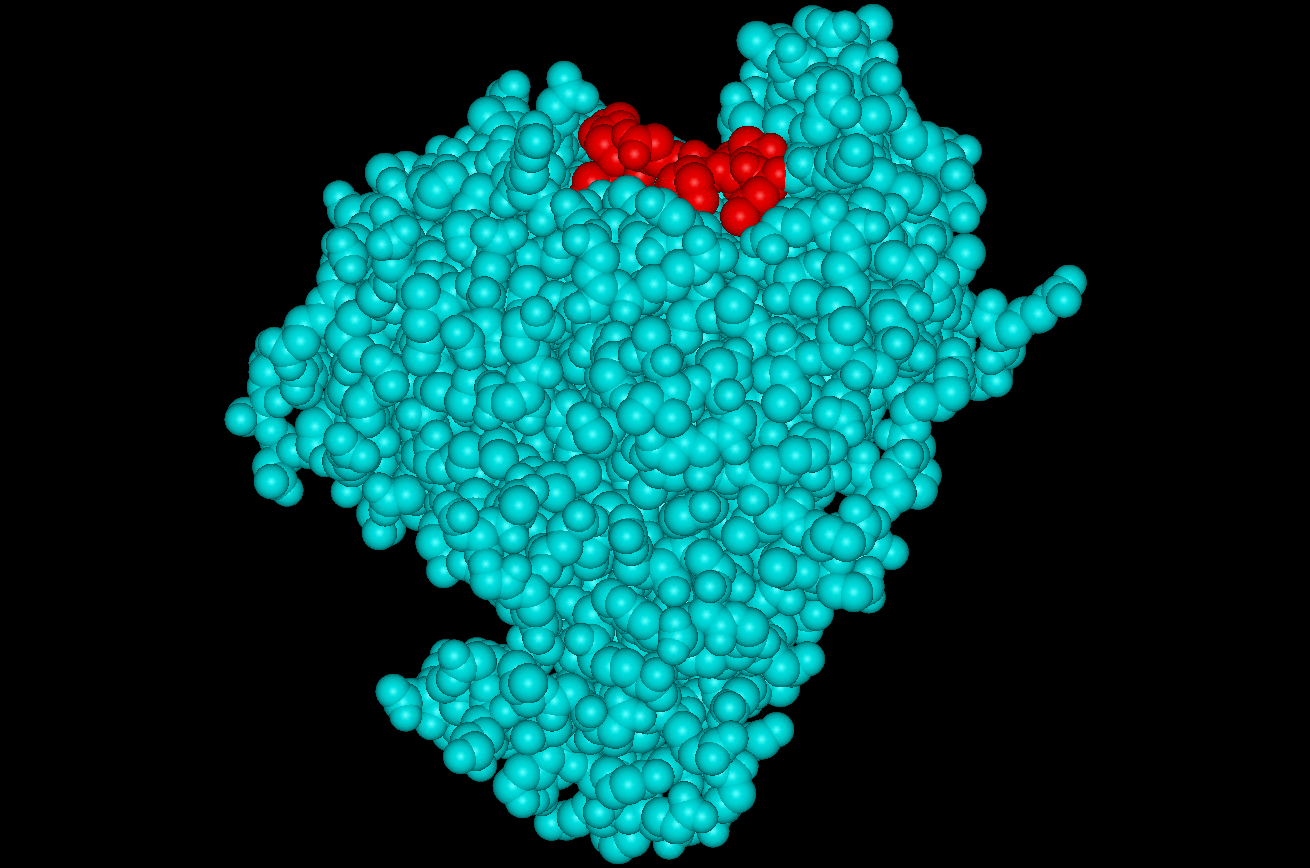 | 6) NS3(_256_EIVDLMCHATFT_267_)  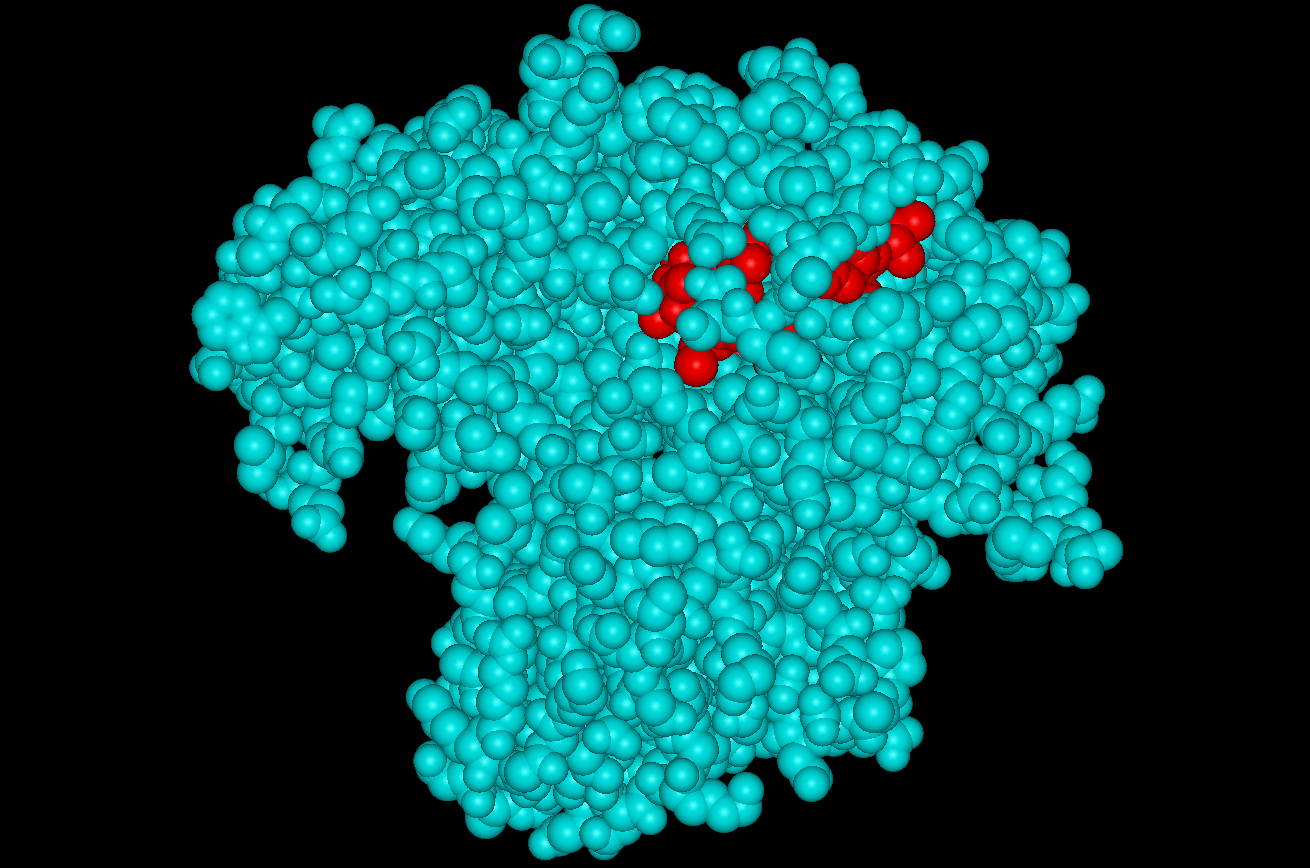 |
| 7) NS3(_284_MDEAHFTDP_292_)  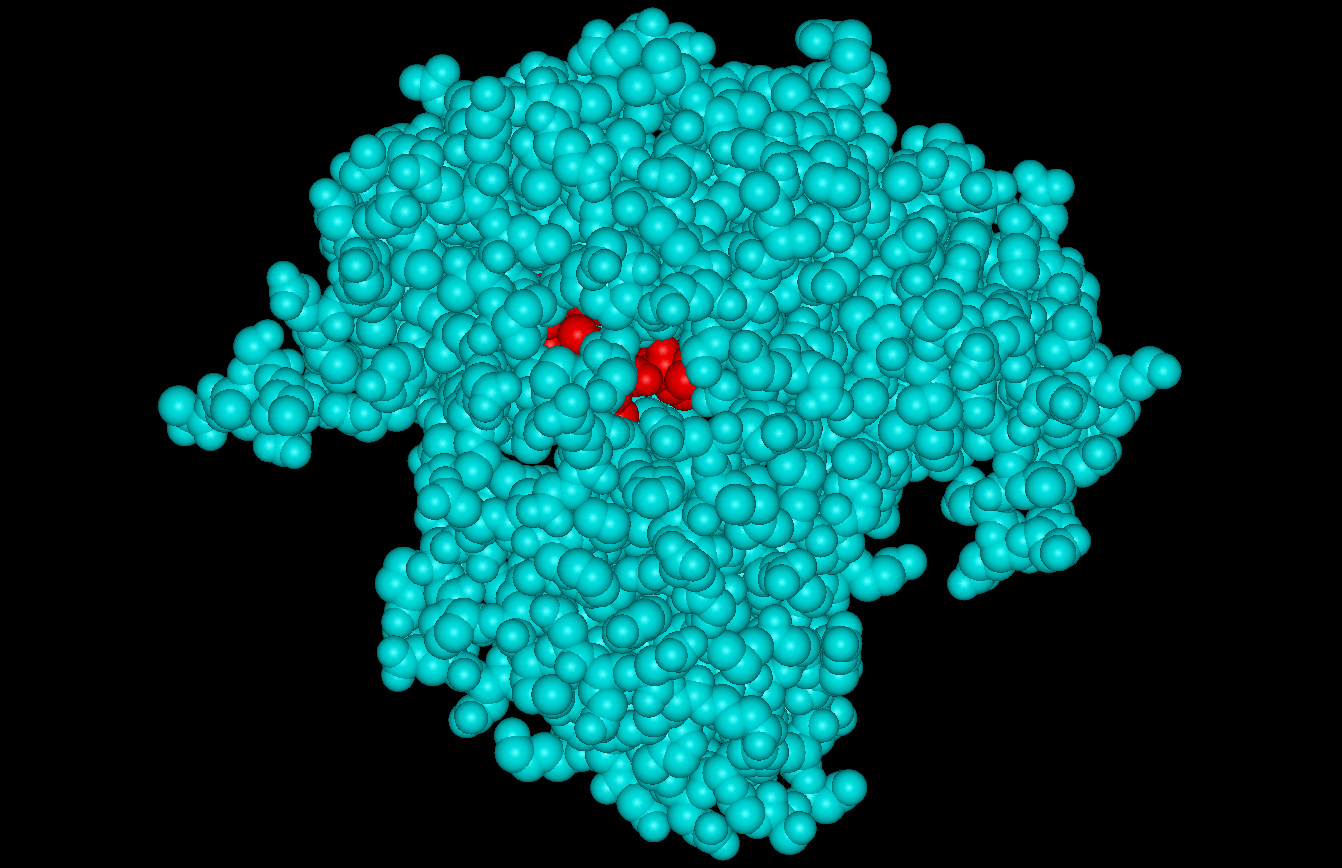 | 8) NS3(_296_AARGYISTRV_305_)  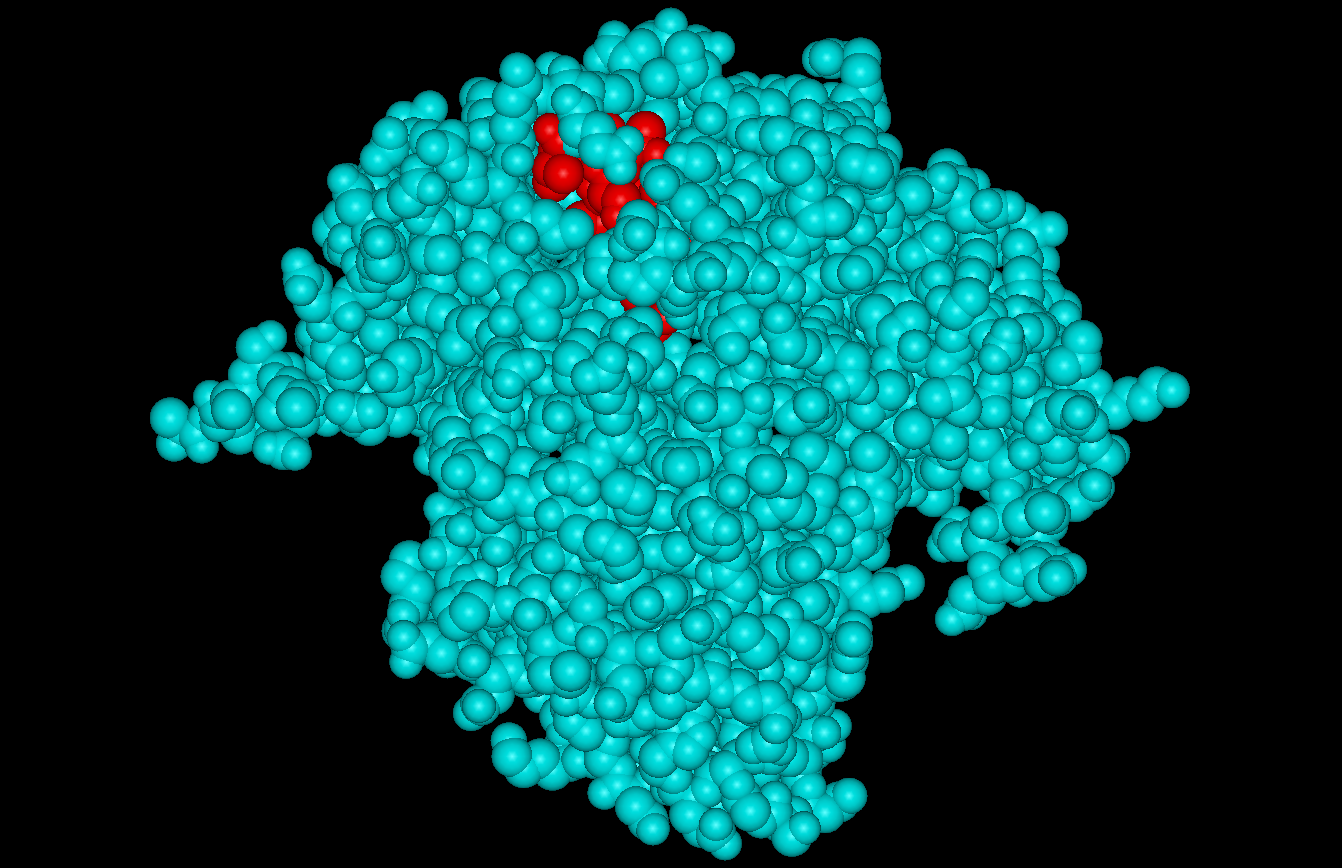 |
| 9) NS3(_313_IFMTATPPG_321_)  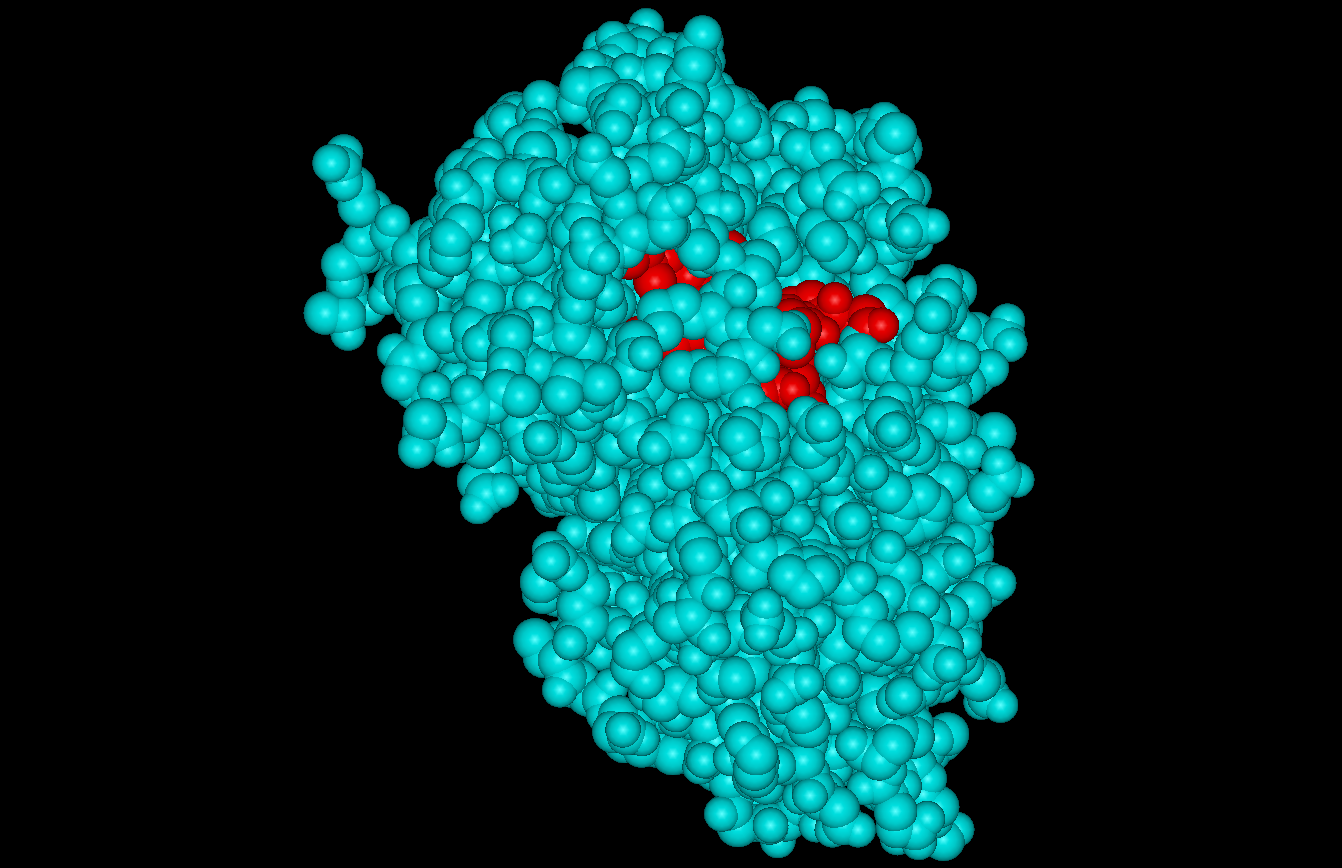 | 10) NS3(_357_GKTVWFVPSIK_367_)  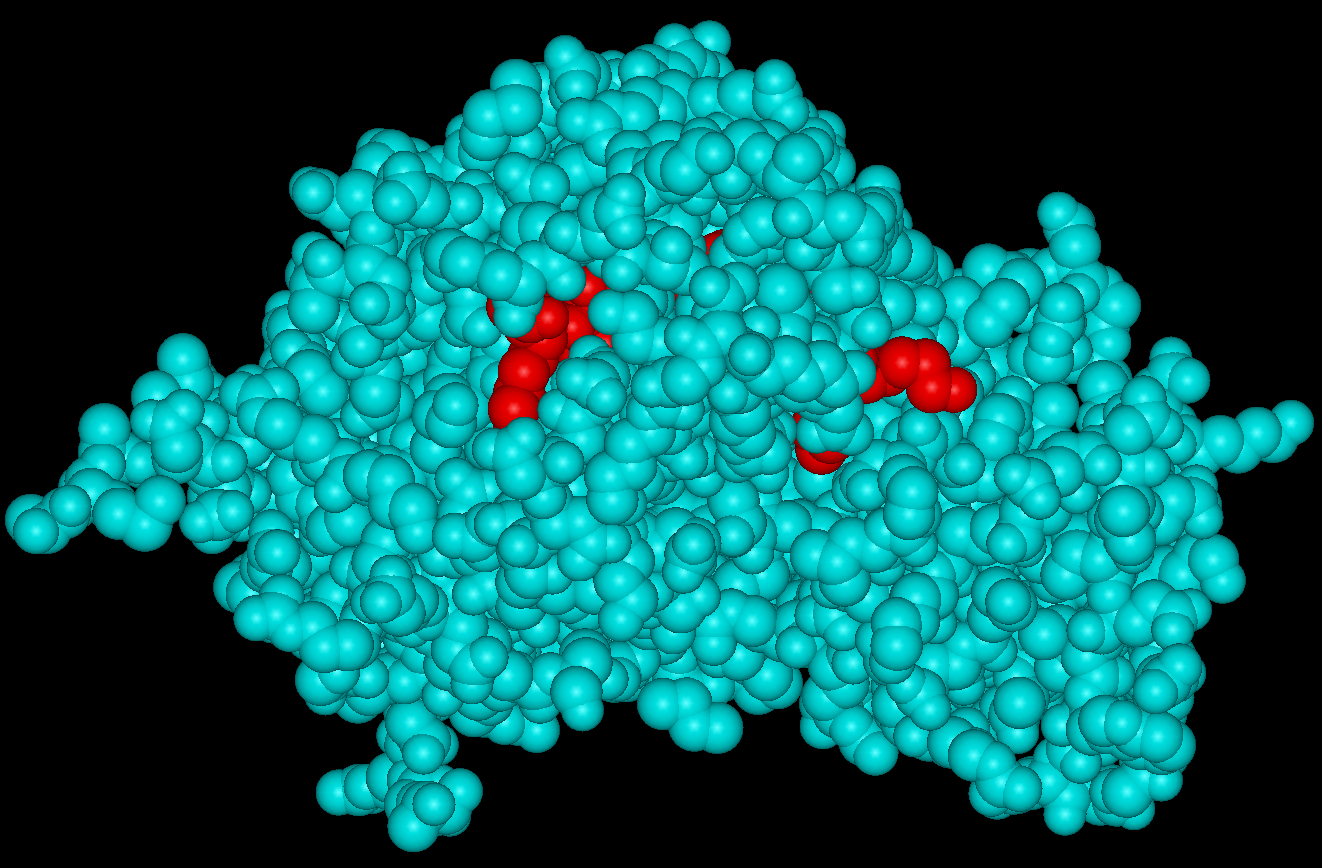 |
| 11) NS3(_383_VIQLSRKTFD_392_)  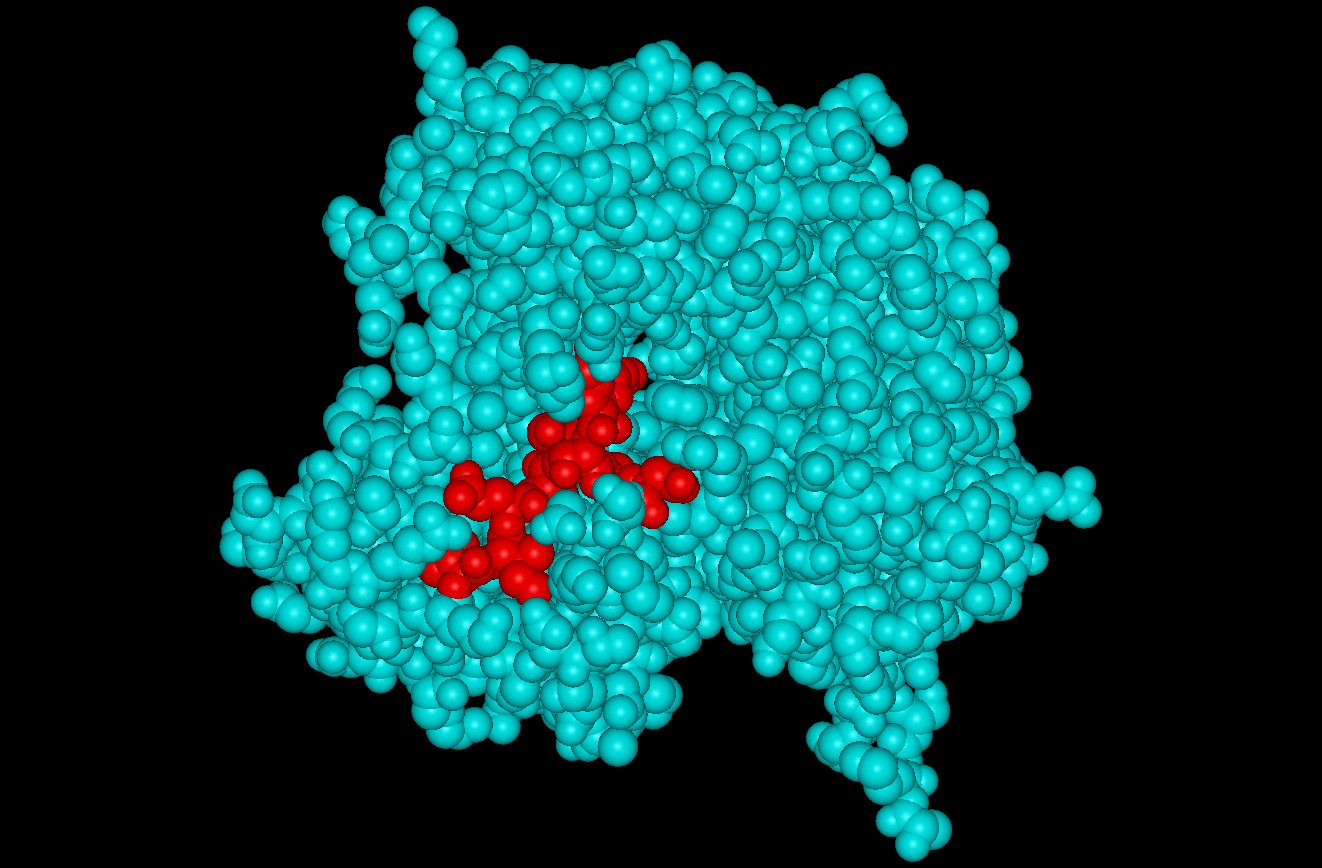 | 12) NS3(_406_VVTTDISEMGANF_418_)  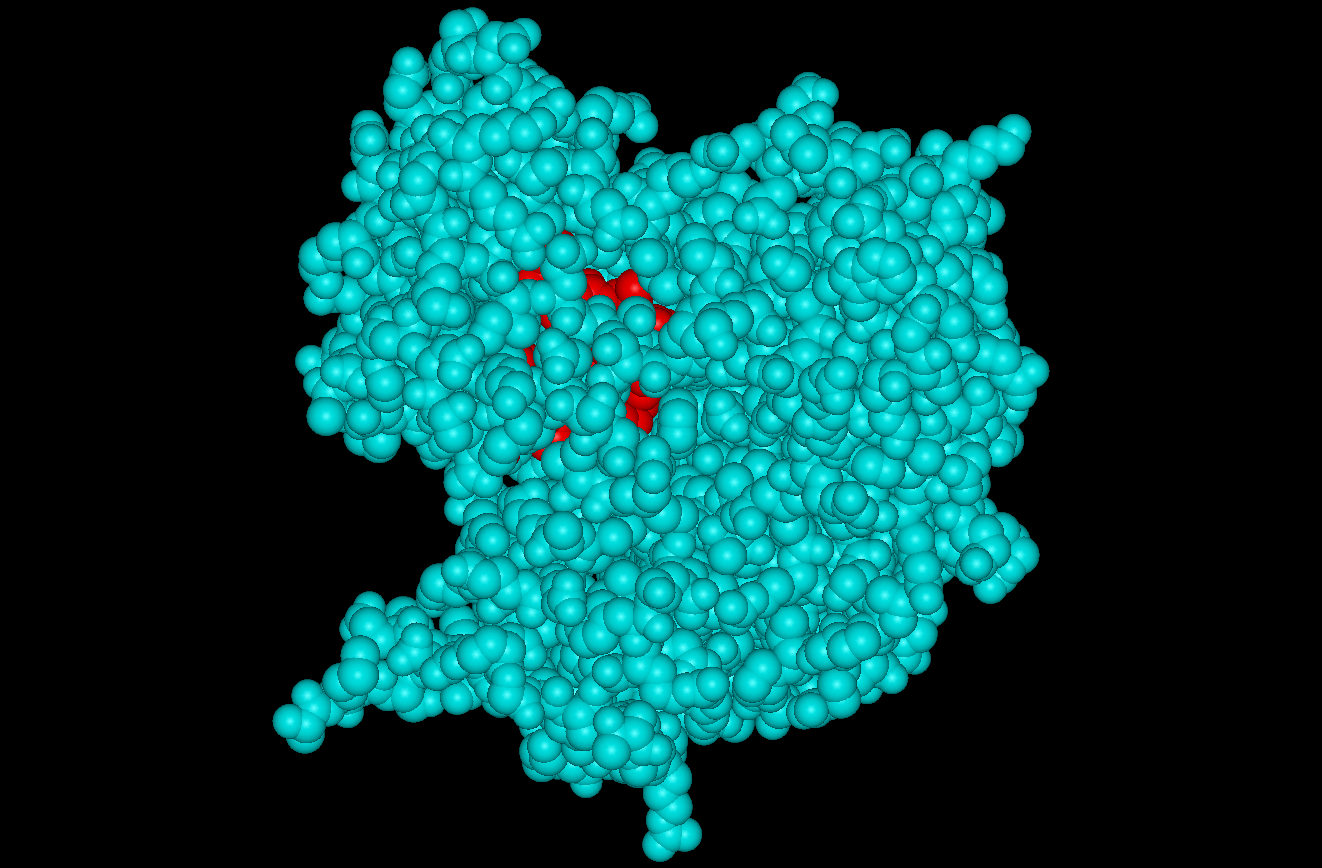 |
| 13) NS3(_491_EAKMLLDNI_499_)  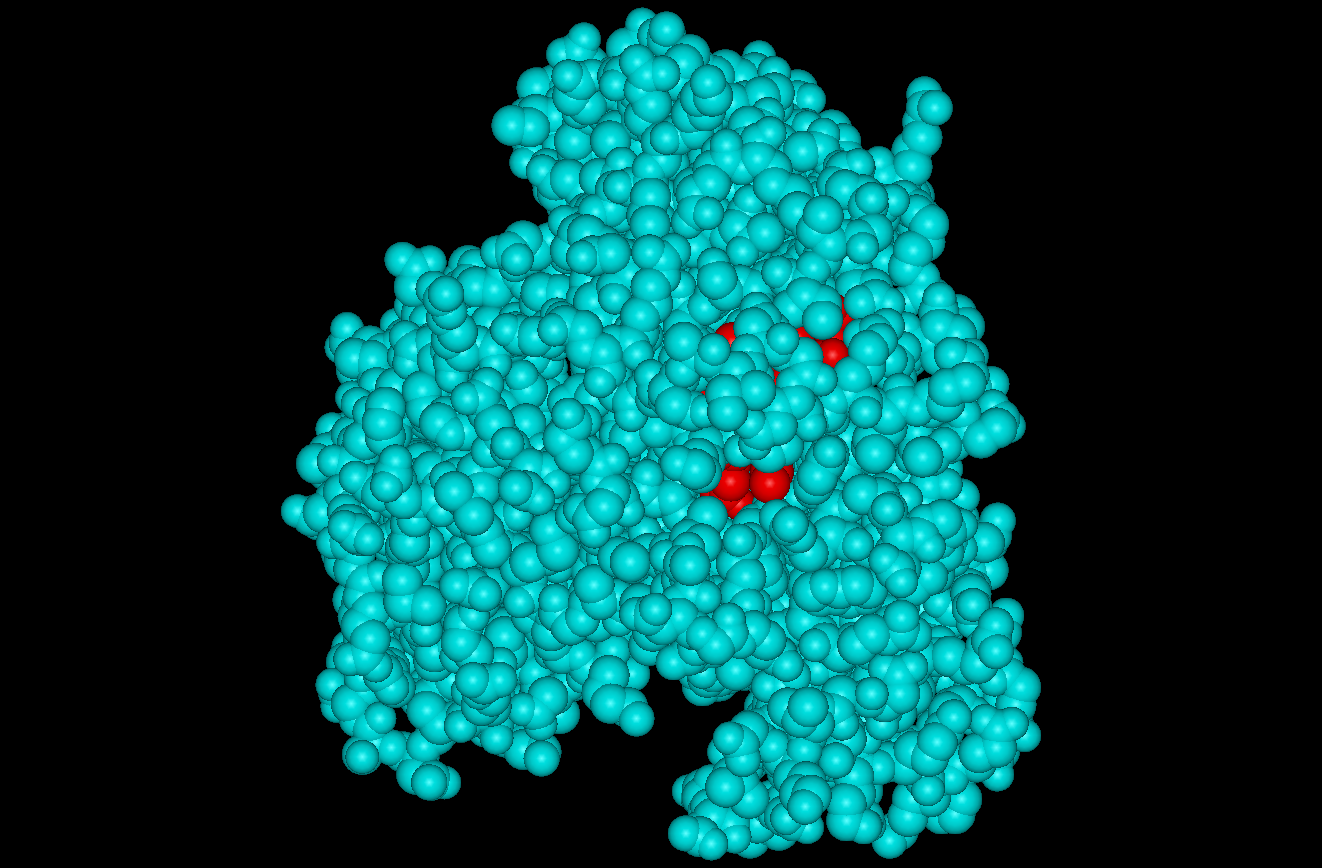 | 14) NS3(_537_LMRRGDLPVWL_547_)  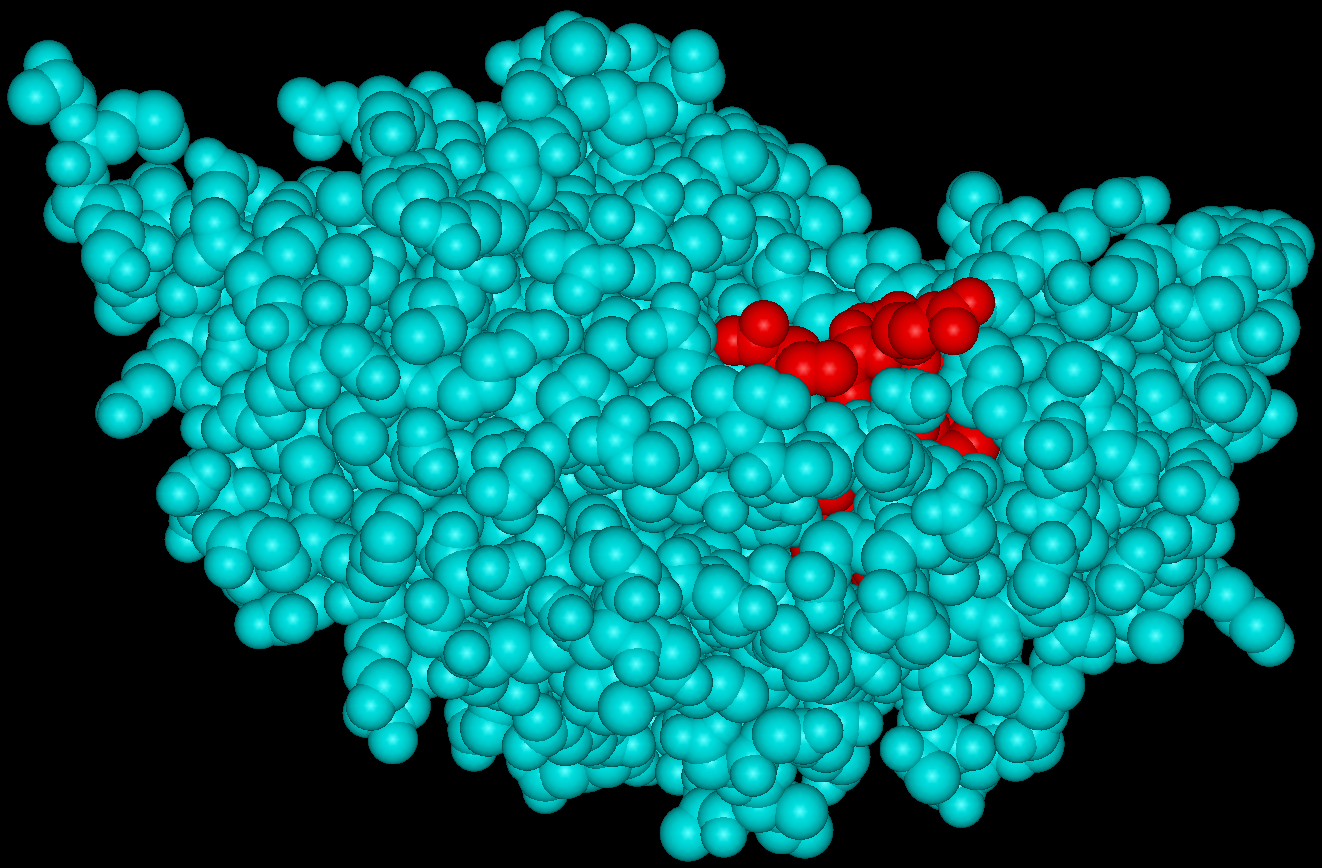 |
| 15) NS5(_6_GETLGEKWK_14_)  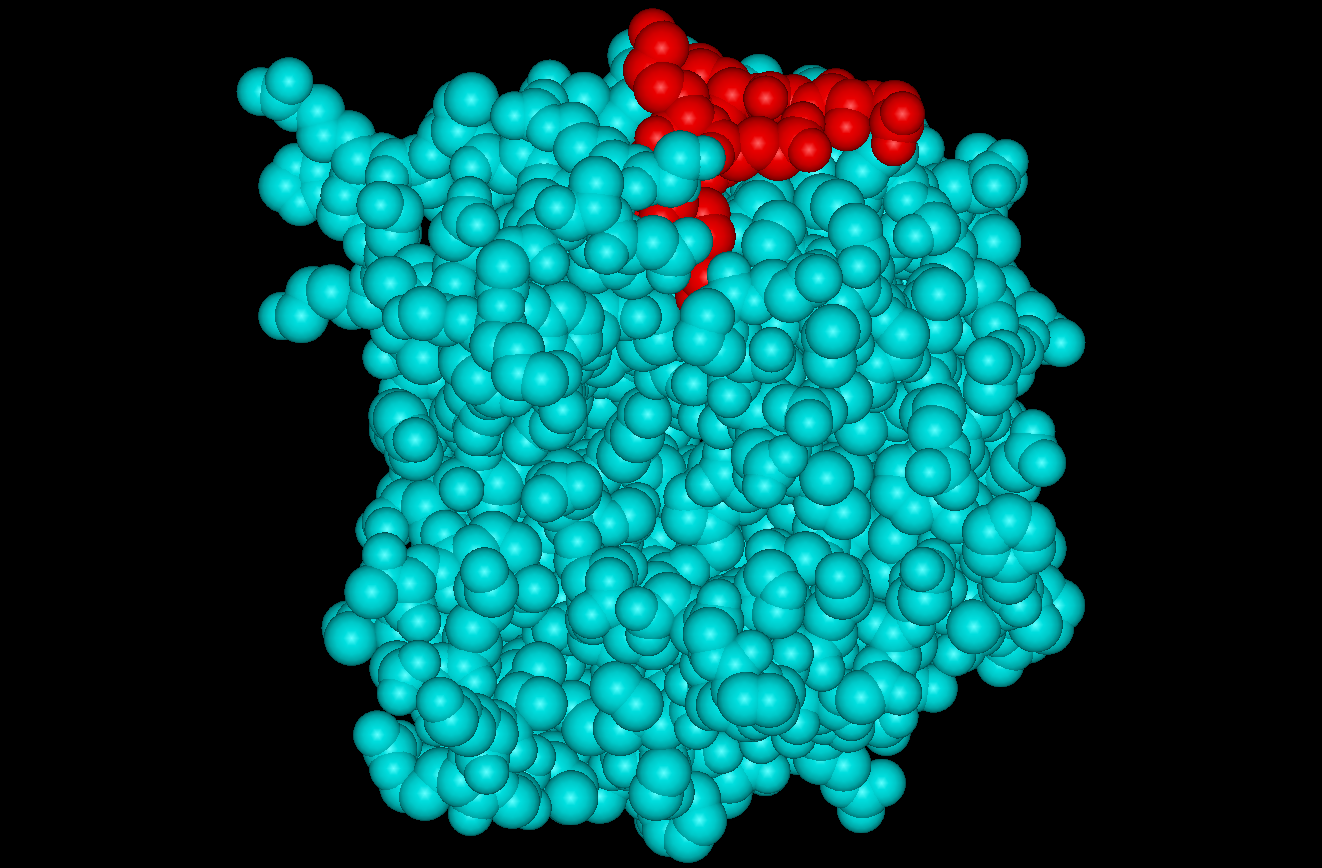 | 16) NS5(_79_DLGCGRGGWSYY_90_)  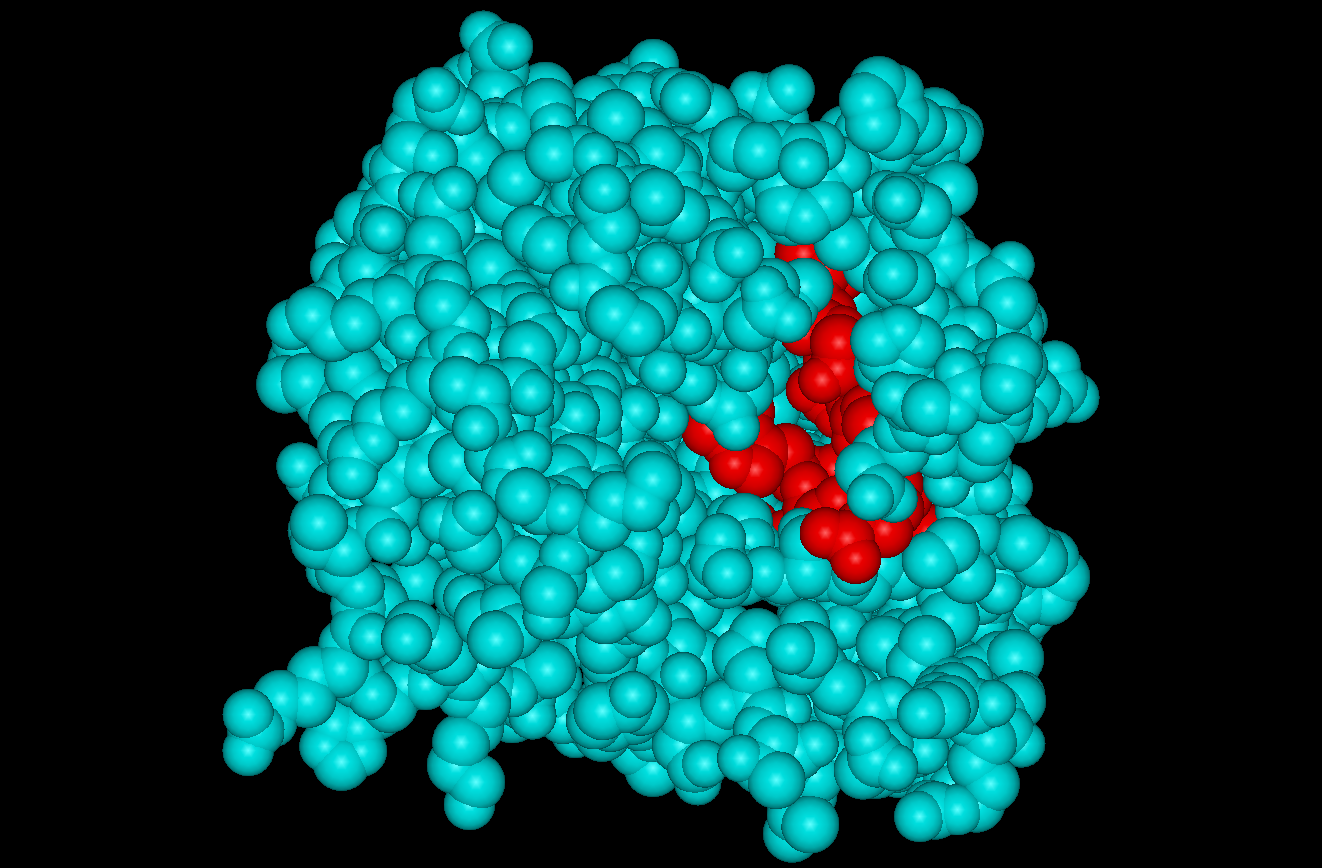 |
| 17) NS5(_104_TKGGPGHEEP_113_)  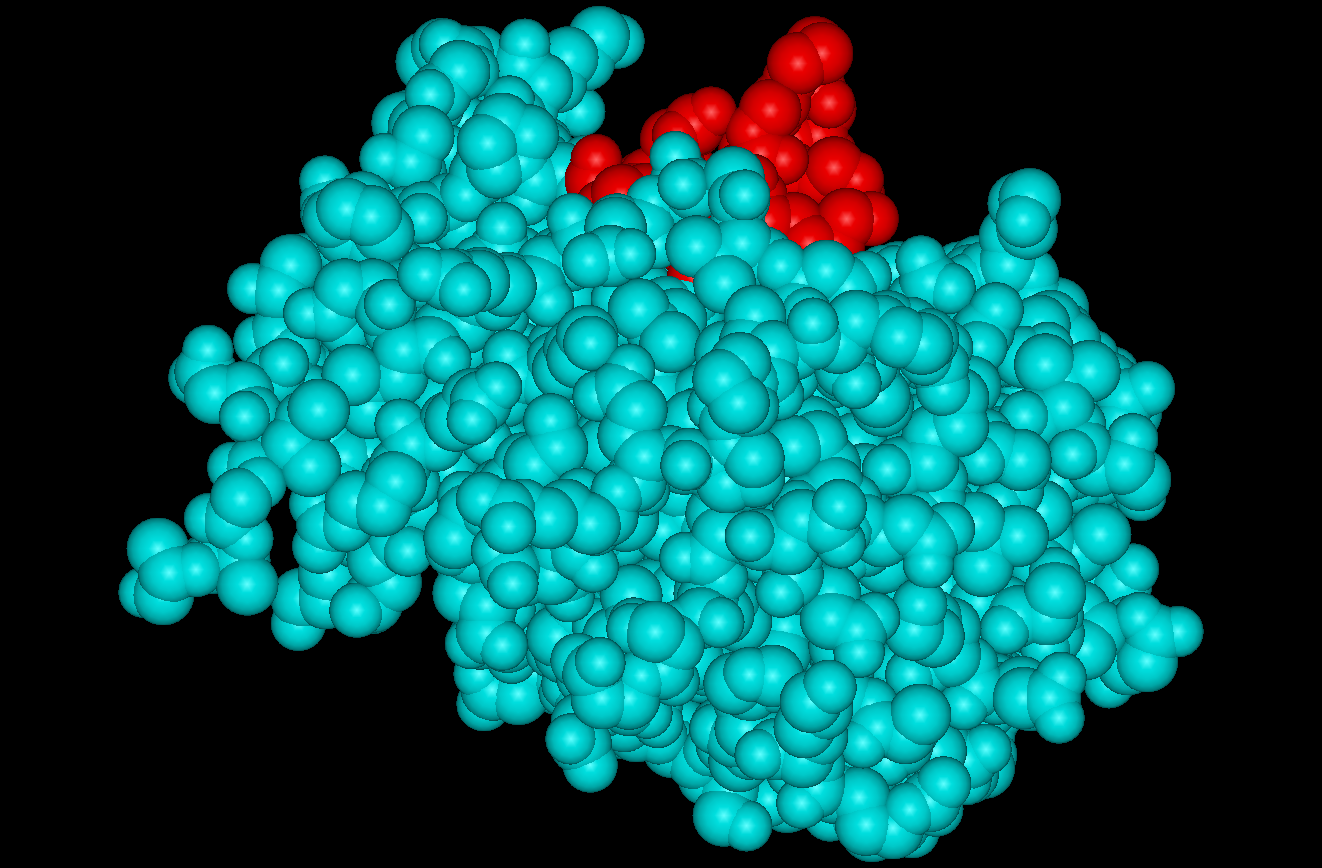 | 18) NS5(_141_DTLLCDIGESS_151_)  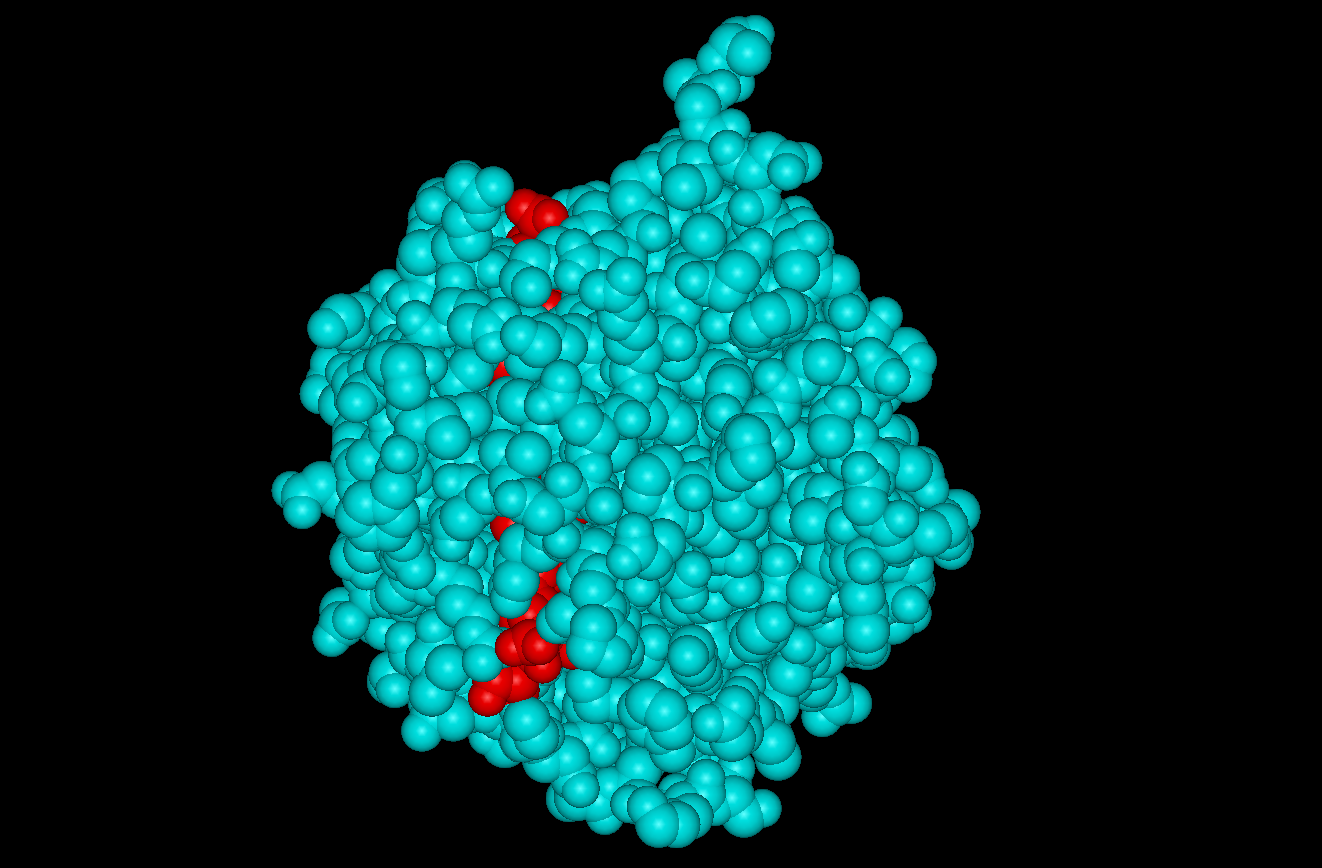 |
| 19) NS5(_209_PLSRNSTHEMYW_220_)  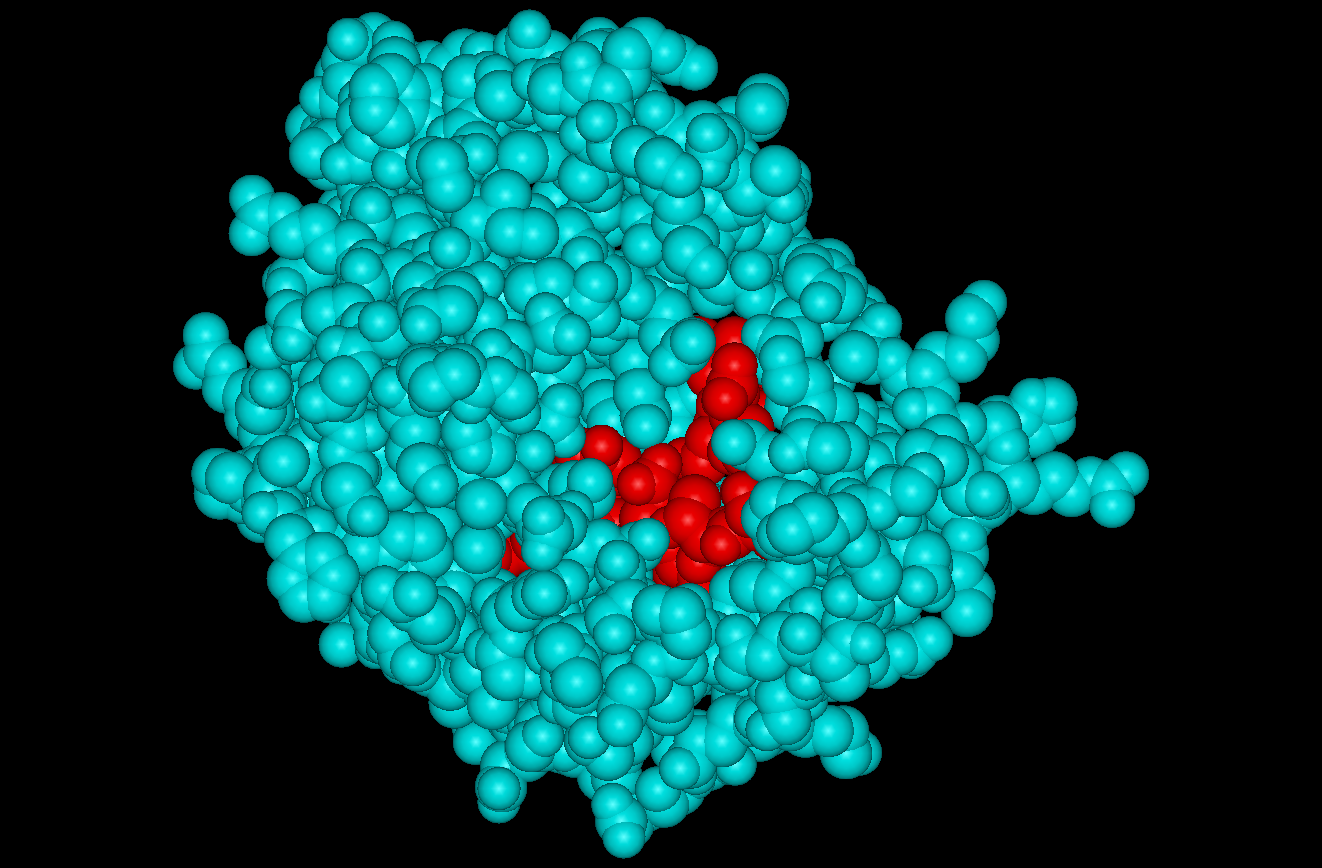 |  |
