# Supplementary material for: Conservation and Variability of Dengue Virus Proteins: Implications for Vaccine Design
Source: PLoS Negl Trop Dis. 2008 Aug 13;2(8):e272. doi: 10.1371/journal.pntd.0000272 (PMC2491585; doi:10.1371/journal.pntd.0000272)
Supplement: Table S1 — The intra-type percentage representation of pan-DENV sequences. (0.10 MB DOC) [file pntd.0000272.s003.doc]

| DENV  protein | Pan-DENV sequence^a^ | % intra-type representation^b^ | | | | | | | |
| --- | --- | --- | --- | --- | --- | --- | --- | --- | --- |
|  |  | DENV-1 | | DENV-2 | | DENV-3 | | DENV-4 | |
|  |  | 2005 | 2007 | 2005 | 2007 | 2005 | 2007 | 2005 | 2007 |
|  |  |  |  |  |  |  |  |  |  |
| E | _97_VDRGWGNGCGLFGKG_111_ | 97.8 | 98.2 | 98.0 | 98.3 | 100.0 | 99.8 | 95.0 | 95.4 |
|  | _252_VLGSQEGAMH_261_ | 95.5 | 96.4 | 98.5 | 98.3 | 98.1 | 98.6 | 99.7 | 99.7 |
|  |  |  |  |  |  |  |  |  |  |
| NS1 | _12_ELKCGSGIF_20_ | 99.5 | 99.2 | 94.9 | 94.3 | 83.1 | 85.9 | 98.6 | 98.7 |
|  | _25_VHTWTEQYKFQ_35_ | 98.6 | 99.0 | 95.1 | 95.3 | 99.0 | 98.6 | 95.0 | 94.8 |
|  | _193_AVHADMGYWIES_204_ | 100.0 | 99.5 | 94.9 | 95.5 | 96.5 | 93.6 | 96.4 | 93.8 |
|  | _229_HTLWSNGVLES_239_ | 96.8 | 97.7 | 95.8 | 96.3 | 96.6 | 98.1 | 100.0 | 97.0 |
|  | _266_GPWHLGKLE_274_ | 100.0 | 100.0 | 94.4 | 92.1 | 99.1 | 99.5 | 100.0 | 100.0 |
|  | _294_RGPSLRTTT_302_ | 93.7 | 95.9 | 99.1 | 99.3 | 98.3 | 98.5 | 100.0 | 100.0 |
|  | _325_GEDGCWYGMEIRP_337_ | 98.1 | 98.0 | 97.0 | 97.1 | 98.2 | 99.0 | 100.0 | 100.0 |
|  |  |  |  |  |  |  |  |  |  |
| NS3 | _46_FHTMWHVTRG_55_ | 100.0 | 100.0 | 100.0 | 100.0 | 100.0 | 100.0 | 100.0 | 100.0 |
|  | _148_GLYGNGVVT_156_ | 100.0 | 100.0 | 99.3 | 99.4 | 100.0 | 100.0 | 100.0 | 100.0 |
|  | _189_LTIMDLHPG_197_ | 100.0 | 100.0 | 98.6 | 98.9 | 100.0 | 100.0 | 100.0 | 97.0 |
|  | _256_EIVDLMCHATFT_267_ | 99.0 | 99.5 | 100.0 | 100.0 | 100.0 | 100.0 | 100.0 | 100.0 |
|  | _284_MDEAHFTDP_292_ | 98.9 | 98.9 | 100.0 | 100.0 | 100.0 | 100.0 | 100.0 | 100.0 |
|  | _296_AARGYISTRV_305_ | 96.7 | 97.7 | 100.0 | 100.0 | 97.3 | 98.1 | 100.0 | 100.0 |
|  | _313_IFMTATPPG_321_ | 100.0 | 99.4 | 100.0 | 100.0 | 100.0 | 100.0 | 100.0 | 100.0 |
|  | _357_GKTVWFVPSIK_367_ | 98.9 | 99.4 | 100.0 | 91.7 | 99.4 | 99.6 | 96.3 | 96.8 |
|  | _383_VIQLSRKTFD_392_ | 81.1 | 89.8 | 98.5 | 98.8 | 98.3 | 98.9 | 100.0 | 100.0 |
|  | _406_VVTTDISEMGANF_418_ | 97.8 | 98.9 | 98.5 | 98.8 | 97.8 | 98.5 | 100.0 | 100.0 |
|  | _491_EAKMLLDNI_499_ | 96.7 | 98.3 | 100.0 | 100.0 | 99.4 | 99.6 | 100.0 | 100.0 |
|  | _537_LMRRGDLPVWL_547_ | 98.9 | 99.4 | 100.0 | 92.2 | 99.4 | 99.2 | 92.6 | 90.3 |
|  |  |  |  |  |  |  |  |  |  |
| NS4a | _126_QRTPQDNQL_134_ | 97.7 | 98.9 | 100.0 | 100.0 | 100.0 | 100.0 | 100.0 | 100.0 |
|  |  |  |  |  |  |  |  |  |  |
| NS4b | _35_PASAWTLYAVATT_47_ | 100.0 | 100.0 | 100.0 | 100.0 | 100.0 | 99.3 | 100.0 | 100.0 |
|  | _118_HYAIIGPGLQAKATREAQKR_137_ | 98.9 | 98.9 | 95.3 | 95.7 | 100.0 | 100.0 | 98.2 | 98.2 |
|  | _139_AAGIMKNPTVDGI_151_ | 95.5 | 97.7 | 97.6 | 97.5 | 97.1 | 98.7 | 100.0 | 100.0 |
|  | _213_FWNTTIAVS_221_ | 97.7 | 98.9 | 100.0 | 100.0 | 100.0 | 100.0 | 98.2 | 98.2 |
|  | _223_ANIFRGSYLAGAGL_236_ | 100.0 | 100.0 | 100.0 | 100.0 | 97.1 | 98.7 | 99.1 | 99.1 |
|  |  |  |  |  |  |  |  |  |  |
| NS5 | _6_GETLGEKWK_14_ | 92.0 | 96.0 | 98.5 | 98.8 | 100.0 | 100.0 | 100.0 | 100.0 |
|  | _79_DLGCGRGGWSYY_90_ | 100.0 | 100.0 | 98.5 | 98.2 | 100.0 | 100.0 | 100.0 | 100.0 |
|  | _104_TKGGPGHEEP_113_ | 90.8 | 94.8 | 98.5 | 98.8 | 100.0 | 100.0 | 100.0 | 100.0 |
|  | _141_DTLLCDIGESS_151_ | 100.0 | 99.4 | 100.0 | 99.4 | 100.0 | 100.0 | 100.0 | 100.0 |
|  | _209_PLSRNSTHEMYW_220_ | 100.0 | 100.0 | 100.0 | 98.8 | 100.0 | 100.0 | 100.0 | 100.0 |
|  | _302_TWAYHGSYE_310_ | 100.0 | 100.0 | 100.0 | 100.0 | 100.0 | 100.0 | 100.0 | 100.0 |
|  | _342_AMTDTTPFGQQRVFKEKVDTRT_363_ | 100.0 | 99.4 | 98.4 | 98.7 | 98.7 | 99.2 | 96.3 | 96.8 |
|  | _450_CVYNMMGKREKKLGEFG_466_ | 100.0 | 99.4 | 92.1 | 93.7 | 96.1 | 97.5 | 100.0 | 100.0 |
|  | _468_AKGSRAIWYMWLGAR_482_ | 96.6 | 98.3 | 100.0 | 100.0 | 98.1 | 98.7 | 100.0 | 100.0 |
|  | _505_SGVEGEGLH_513_ | 100.0 | 100.0 | 95.3 | 95.7 | 98.7 | 98.8 | 100.0 | 100.0 |
|  | _531_YADDTAGWDTRIT_543_ | 97.7 | 98.9 | 100.0 | 100.0 | 99.4 | 99.6 | 100.0 | 100.0 |
|  | _568_IFKLTYQNKVV_578_ | 100.0 | 99.4 | 96.9 | 95.7 | 96.9 | 97.9 | 100.0 | 100.0 |
|  | _597_DQRGSGQVGTYGLNTFTNME_616_ | 95.4 | 93.1 | 83.1 | 81.0 | 97.5 | 98.3 | 96.3 | 96.8 |
|  | _658_RMAISGDDCVVKP_670_ | 100.0 | 100.0 | 100.0 | 100.0 | 95.6 | 97.1 | 100.0 | 100.0 |
|  | _707_VPFCSHHFH_715_ | 97.7 | 98.9 | 100.0 | 100.0 | 100.0 | 100.0 | 96.3 | 96.8 |
|  | _765_LMYFHRRDLRLA_776_ | 100.0 | 100.0 | 98.5 | 98.8 | 98.1 | 98.8 | 100.0 | 100.0 |
|  | _790_PTSRTTWSIHA_800_ | 98.9 | 98.3 | 98.4 | 98.8 | 98.1 | 98.7 | 100.0 | 100.0 |
|  |  |  |  |  |  |  |  |  |  |

^a^ Amino acid positions numbered according to the sequence alignments of the 4 DENV types

^b^ Rounded to 1 decimal place
