# Supplementary material for: Conservation and Variability of Dengue Virus Proteins: Implications for Vaccine Design
Source: PLoS Negl Trop Dis. 2008 Aug 13;2(8):e272. doi: 10.1371/journal.pntd.0000272 (PMC2491585; doi:10.1371/journal.pntd.0000272)
Supplement: Table S2 — Pan-DENV sequences, entropy and representation of variants. (0.08 MB DOC) [file pntd.0000272.s004.doc]

| DENV  protein | Pan-DENV sequence^a^ | Peptide entropy^b^ | | % variant representation^c^ | |
| --- | --- | --- | --- | --- | --- |
|  |  | 2005 | 2007 | 2005 | 2007 |
|  |  |  |  |  |  |
| E | _97_VDRGWGNGCGLFGKG_111_ | 0.2 | 0.2 | 1-2 | 1 |
|  | _252_VLGSQEGAMH_261_ | 0.2 | 0.2 | 1-2 | 1-2 |
|  |  |  |  |  |  |
| NS1 | _12_ELKCGSGIF_20_ | 0.5 | 0.5 | 5 | 5 |
|  | _25_VHTWTEQYKFQ_35_ | 0.3 | 0.3 | 3 | 2-3 |
|  | _193_AVHADMGYWIES_204_ | 0.3 | 0.3 | 2-3 | 2-3 |
|  | _229_HTLWSNGVLES_239_ | 0.3 | 0.3 | 3 | 2-3 |
|  | _266_GPWHLGKLE_274_ | 0.2 | 0.3 | 3 | 3 |
|  | _294_RGPSLRTTT_302_ | 0.2 | 0.2 | 3 | 2 |
|  | _325_GEDGCWYGMEIRP_337_ | 0.2 | 0.2 | < 1-2 | < 1-2 |
|  |  |  |  |  |  |
| NS3 | _46_FHTMWHVTRG_55_ | 0.0 | 0.0 | 0 | 0 |
|  | _148_GLYGNGVVT_156_ | < 0.1 | < 0.1 | < 1 | < 1 |
|  | _189_LTIMDLHPG_197_ | 0.1 | 0.1 | 1 | 1 |
|  | _256_EIVDLMCHATFT_267_ | < 0.1 | < 0.1 | < 1 | < 1 |
|  | _284_MDEAHFTDP_292_ | < 0.1 | 0.1 | < 1 | < 1 |
|  | _296_AARGYISTRV_305_ | 0.2 | 0.2 | 2 | 1 |
|  | _313_IFMTATPPG_321_ | 0.0 | < 0.1 | 0 | < 1 |
|  | _357_GKTVWFVPSIK_367_ | 0.1 | 0.2 | < 1-1 | 1-2 |
|  | _383_VIQLSRKTFD_392_ | 0.4 | 0.3 | 5 | 4 |
|  | _406_VVTTDISEMGANF_418_ | 0.2 | 0.1 | 1-2 | 1 |
|  | _491_EAKMLLDNI_499_ | 0.1 | 0.1 | 1 | 1 |
|  | _537_LMRRGDLPVWL_547_ | 0.1 | 0.2 | 1 | 3 |
|  |  |  |  |  |  |
| NS4a | _126_QRTPQDNQL_134_ | 0.1 | < 0.1 | 1 | < 1 |
|  |  |  |  |  |  |
| NS4b | _35_PASAWTLYAVATT_47_ | 0.0 | < 0.1 | 0 | < 1 |
|  | _118_HYAIIGPGLQAKATREAQKR_137_ | 0.2 | 0.2 | 1-2 | 1 |
|  | _139_AAGIMKNPTVDGI_151_ | 0.2 | 0.2 | 2 | 1-2 |
|  | _213_FWNTTIAVS_221_ | 0.1 | 0.1 | 1 | 1 |
|  | _223_ANIFRGSYLAGAGL_236_ | 0.1 | < 0.1 | 0-1 | 0- < 1 |
|  |  |  |  |  |  |
| NS5 | _6_GETLGEKWK_14_ | 0.2 | 0.2 | 2 | 2 |
|  | _79_DLGCGRGGWSYY_90_ | 0.1 | 0.1 | 1 | < 1-1 |
|  | _104_TKGGPGHEEP_113_ | 0.2 | 0.2 | 3 | 2 |
|  | _141_DTLLCDIGESS_151_ | 0.0 | < 0.1 | 0 | 0- < 1 |
|  | _209_PLSRNSTHEMYW_220_ | 0.0 | 0.1 | 0 | < 1 |
|  | _302_TWAYHGSYE_310_ | 0.0 | 0.0 | 0 | 0 |
|  | _342_AMTDTTPFGQQRVFKEKVDTRT_363_ | 0.1 | 0.1 | 0-1 | 0-1 |
|  | _450_CVYNMMGKREKKLGEFG_466_ | 0.3 | 0.2 | 1-3 | 1-2 |
|  | _468_AKGSRAIWYMWLGAR_482_ | 0.1 | 0.1 | < 1-1 | < 1-1 |
|  | _505_SGVEGEGLH_513_ | 0.2 | 0.2 | 2 | 2 |
|  | _531_YADDTAGWDTRIT_543_ | 0.1 | 0.1 | < 1-1 | < 1-1 |
|  | _568_IFKLTYQNKVV_578_ | 0.2 | 0.2 | 2 | 2 |
|  | _597_DQRGSGQVGTYGLNTFTNME_616_ | 0.4 | 0.3 | 1-5 | 1-5 |
|  | _658_RMAISGDDCVVKP_670_ | 0.2 | 0.1 | 1-2 | 1 |
|  | _707_VPFCSHHFH_715_ | 0.1 | 0.1 | 1 | 1 |
|  | _765_LMYFHRRDLRLA_776_ | 0.1 | 0.1 | 1 | 1 |
|  | _790_PTSRTTWSIHA_800_ | 0.2 | 0.1 | 1-2 | 1 |
|  |  |  |  |  |  |

^a^ Amino acid positions numbered according to the sequence alignments of the 4 DENV types

^b^ Maximum nonamer peptide entropy across all DENV sequences (rounded to 1 decimal place)

^c^ Minimum and maximum percentage representation of nonamer variants in all DENV sequences (rounded to whole number)
