# Supplementary material for: Conservation and Variability of Dengue Virus Proteins: Implications for Vaccine Design
Source: PLoS Negl Trop Dis. 2008 Aug 13;2(8):e272. doi: 10.1371/journal.pntd.0000272 (PMC2491585; doi:10.1371/journal.pntd.0000272)
Supplement: Table S3 — Functional and structural properties of pan-DENV sequences. (0.06 MB DOC) [file pntd.0000272.s005.doc]

| DENV  protein | Pan-DENV sequence^a^ | Functional domains and motifs^b^ | Putative post-transcriptional modifications^b^ |
| --- | --- | --- | --- |
|  |  |  |  |
| E | _97_VDRGWGNGCGLFGKG_111_ | Dimerisation Domain, Fusion Peptide | N-Myristoylation |
|  | _252_VLGSQEGAMH_261_ | Dimerisation Domain | - |
|  |  |  |  |
| NS1 | _12_ELKCGSGIF_20_ | - | N-Myristoylation |
|  | _25_VHTWTEQYKFQ_35_ | - | CKII |
|  | _294_RGPSLRTTT_302_ | - | PKC |
|  | _325_GEDGCWYGMEIRP_337_ | - | N-Myristoylation |
|  |  |  |  |
| NS3 | _46_FHTMWHVTRG_55_ | Peptidase S7 | - |
|  | _148_GLYGNGVVT_156_ | - | N-Myristoylation |
|  | _189_LTIMDLHPG_197_ | - | CKII |
|  | _256_EIVDLMCHATFT_267_ | DEAD/H Domain | *-* |
|  | _284_MDEAHFTDP_292_ | DEAD/H Domain | - |
|  | _296_AARGYISTRV_305_ | Microbodies C-Terminal Targeting Signal | PKC |
|  | _313_IFMTATPPG_321_ | DEAD/H Domain | *-* |
|  | _357_GKTVWFVPSIK_367_ | - | PKC |
|  | _383_VIQLSRKTFD_392_ | - | PKC |
|  | _537_LMRRGDLPVWL_547_ | Cell Attachment | - |
|  |  |  |  |
| NS4a | _126_QRTPQDNQL_134_ | - | CKII |
|  |  |  |  |
| NS4b | _213_FWNTTIAVS_221_ | - | N-Glycosylation |
|  | _223_ANIFRGSYLAGAGL_236_ | - | N-Myristoylation |
|  |  |  |  |
| NS5 | _6_GETLGEKWK_14_ | - | CKII |
|  | _79_DLGCGRGGWSYY_90_ | FtsJ-like Methyltransferase Domain | N-Myristoylation |
|  | _209_PLSRNSTHEMYW_220_ | - | N-Glycosylation, CKII |
|  | _450_CVYNMMGKREKKLGEFG_466_ | - | Amidation |
|  | _505_SGVEGEGLH_513_ | - | CKII |
|  | _597_DQRGSGQVGTYGLNTFTNME_616_ | RdRp Catalytic Domain | N-Myristoylation, CKII |
|  | _658_RMAISGDDCVVKP_670_ | RdRp Catalytic Domain | CKII |
|  | _790_PTSRTTWSIHA_800_ | - | PKC |
|  |  |  |  |

^a^ Amino acid positions numbered according to the sequence alignments of the 4 DENV types

^b^ Described in the literature and/or identified using the Prosite [30] and Pfam [32] databases. Prosite (PS) and Pfam (PF) accession numbers: PS00001, N-Glycosylation; PS00005, Protein Kinase II Phosphorylation (PKC); PS00006, Casein Kinase II Phosphorylation (CKII); PS00008, N-Myristoylation; PS00009, Amidation; PS00016, Cell Attachment; PS00342, Microbodies C-terminal Targeting Signal; PS50507, RNA-dependent RNA polymerase (RdRp) Catalytic Domain; PF00869, Dimerisation Domain; PF00949, Peptidase S7; PF01728, FtsJ-like Methyltransferase; PF07652, Flavivirus DEAD/H Domain.
