# Supplementary material for: Conservation and Variability of Dengue Virus Proteins: Implications for Vaccine Design
Source: PLoS Negl Trop Dis. 2008 Aug 13;2(8):e272. doi: 10.1371/journal.pntd.0000272 (PMC2491585; doi:10.1371/journal.pntd.0000272)
Supplement: Table S4 — Distribution of pan-DENV sequences in nature. (0.12 MB DOC) [file pntd.0000272.s006.doc]

| DENV  protein | Pan-DENV sequence^a^ | Species (#)^b^ | Percentage representation (%) | number of sequence analyzed^c^ | | | | | | | | | | | | | |
| --- | --- | --- | --- | --- | --- | --- | --- | --- | --- | --- | --- | --- | --- | --- | --- | --- |
|  |  |  | LEV | WNV | JEV | MVE | UV | KBV | IH | TBEV | LV | OMSK | LIV | PV | KFDV | YFV |
|  |  |  |  |  |  |  |  |  |  |  |  |  |  |  |  |  |
| E | _97_VDRGWGNGCGLFGKG_111_ | 19 | 97|77 | 97|276 | 97|250 |  |  |  |  |  |  |  |  |  |  | 82|179 |
|  |  |  |  |  |  |  |  |  |  |  |  |  |  |  |  |  |
| NS1 | _12_ELKCGSGIF_20_ | 2 |  |  |  |  |  |  |  |  |  |  |  |  |  |  |
|  | _294_RGPSLRTTT_302_ | 3 |  |  | 3\|58 |  |  |  |  |  |  |  |  |  |  |  |
|  | _325_GEDGCWYGMEIRP_337_ | 10 | 90\|30 | 96\|138 | 31\|186 |  |  |  |  |  |  |  |  |  |  |  |
|  |  |  |  |  |  |  |  |  |  |  |  |  |  |  |  |  |
| NS3 | _46_FHTMWHVTRG_55_ | 23 |  |  |  |  |  |  |  | 89|19 |  |  |  |  |  | 87|23 |
|  | _256_EIVDLMCHATFT_267_ | 3 |  |  |  |  |  |  |  |  |  |  |  |  |  |  |
|  | _284_MDEAHFTDP_292_ | 10 |  | 99\|134 | 98\|54 |  |  |  |  |  |  |  |  |  |  |  |
|  | _296_AARGYISTRV_305_ | 3 | 9\|98 | 4\|137 |  |  |  |  |  |  |  |  |  |  |  |  |
|  | _313_IFMTATPPG_321_ | 11 | 96\|26 | 100\|134 | 98\|53 |  |  |  |  |  |  |  |  |  |  |  |
|  | _357_GKTVWFVPSIK_367_ | 8 |  | 94\|141 |  |  |  |  | 17\|12 |  |  |  |  |  |  |  |
|  | _383_VIQLSRKTFD_392_ | 1 |  |  |  |  |  |  |  |  |  |  |  |  |  |  |
|  | _406_VVTTDISEMGANF_418_ | 35 | 34\|77 | 91\|146 | 21\|248 |  |  |  |  | 68\|25 |  |  |  |  |  |  |
|  | _491_EAKMLLDNI_499_ | 1 |  |  |  |  |  |  |  |  |  |  |  |  |  |  |
|  |  |  |  |  |  |  |  |  |  |  |  |  |  |  |  |  |
| NS4b | _223_ANIFRGSYLAGAGL_236_ | 1 |  |  |  |  |  |  |  |  |  |  |  |  |  |  |
|  |  |  |  |  |  |  |  |  |  |  |  |  |  |  |  |  |
| NS5 | _6_GETLGEKWK_14_ | 4 |  |  |  |  |  |  |  |  |  |  |  |  |  |  |
|  | _79_DLGCGRGGWSYY_90_ | 37 | 77\|35 | 96\|140 | 20\|244 |  |  |  |  | 82\|38 |  |  |  | 83\|18 | 40\|10 | 78\|27 |
|  | _104_TKGGPGHEEP_113_ | 9 | 93\|28 | 89\|148 |  |  |  |  |  |  |  |  |  |  |  |  |
|  | _141_DTLLCDIGESS_151_ | 13 |  | 100\|134 | < 1\|268 |  |  |  |  |  |  |  |  |  |  | 84\|25 |
|  | _209_PLSRNSTHEMYW_220_ | 31 | 90\|29 | 100\|134 |  |  |  |  |  | 61\|28 |  |  |  | 100\|17 |  | 95\|22 |
|  | _302_TWAYHGSYE_310_ | 1 |  |  |  |  |  |  |  |  |  |  |  |  |  |  |
|  | _342_AMTDTTPFGQQRVFKEKVDTRT_363_ | 29 | 33\|79 | 50\|272 | 18\|289 | 18\|17 | 23\|13 |  | 25\|12 | 32\|34 |  |  |  | 60\|45 |  | 51\|41 |
|  | _450_CVYNMMGKREKKLGEFG_466_ | 35 | 24\|103 | 33\|344 | 69\|74 | 20\|15 |  | 7\|14 | 14\|14 | 63\|27 |  |  | 11\|19 | 41\|41 |  | 23\|90 |
|  | _468_AKGSRAIWYMWLGAR_482_ | 44 | 32\|19 |  |  |  |  |  | 62\|13 | 82\|22 | 42\|12 |  |  | 41\|41 |  | 11\|208 |
|  | _531_YADDTAGWDTRIT_543_ | 59 | 90\|30 | 73\|193 | 88\|58 |  |  |  | 80\|10 | 69\|26 |  | 93\|14 |  | 84\|19 |  | 85\|26 |
|  | _658_RMAISGDDCVVKP_670_ | 27 | 73\|37 | 50\|272 | 95\|55 | 50\|10 |  |  |  |  |  |  |  | 47\|34 |  | 3\|37 |
|  | _707_VPFCSHHFH_715_ | 33 |  |  |  |  |  |  |  | 100\|19 |  | 100\|13 |  | 76\|17 |  | 95\|22 |
|  | _765_LMYFHRRDLRLA_776_ | 37 | 64\|42 | 91\|151 | 83\|59 |  |  |  | 44\|18 |  |  |  |  |  |  |  |
|  |  |  |  |  |  |  |  |  |  |  |  |  |  |  |  |  |

^a^ Amino acid positions numbered according to the sequence alignments of the 4 DENV types

^b^ Species (#) column indicates the number of viral species that shared at least 9 consecutive amino acids of the pan-DENV sequence

^c^ Percentage representation (rounded to whole number) of the pan-DENV sequences is only shown for viral species with ≥ 10 total sequences reported at the NCBI Entrez protein database. These viral species included: LEV, *St. Louis encephalitis virus*; WNV, *West Nile virus*; JEV, *Japanese encephalitis virus*; MVE, *Murray Valley encephalitis virus*; UV, *Usutu virus*; KBV, *Kokobera virus*; IH, *Ilheus virus*; TBEV, *Tick-born encephalitis virus*; LV, *Langat virus*; OMSK, *Omsk hemorrhagic fever virus*; LIV, *Louping ill* *virus*; PV, *Powassan virus*; KDFV, *Kyasanur forest disease* *virus*; and YFV, *Yellow fever virus*. However, despite having a total of ≥ 10 sequences reported, some of these viruses had less than 10 of the relevant conserved sequence (indicated by cells shaded in grey). Empty cells indicate no match between the pan-DENV sequences and the *Flavivirus*.
