# Supplementary material for: Conservation and Variability of Dengue Virus Proteins: Implications for Vaccine Design
Source: PLoS Negl Trop Dis. 2008 Aug 13;2(8):e272. doi: 10.1371/journal.pntd.0000272 (PMC2491585; doi:10.1371/journal.pntd.0000272)
Supplement: Table S5 — Candidate putative HLA supertype-restricted binding nonamer peptides in pan-DENV sequences, predicted by immunoinformatic algorithms. (0.20 MB DOC) [file pntd.0000272.s007.doc]

| DENV  protein | Pan-DENV sequence and the predicted HLA supertype-restricted binding nonamer(s) ^a^ | HLA supertype-restriction of predicted nonamer peptide^b^ | | | | |
| --- | --- | --- | --- | --- | --- | --- |
|  |  | Class I^c^ | | | Class II^d^ | |
|  |  | NetCTL | Multipred | ARB | Multipred | TEPITOPE |
|  |  |  |  |  |  |  |
| E | _97_VDRGWGNGCGLFGKG_111_ |  |  |  |  |  |
|  | _99_RGWGNGCGL_107_ | B7 | - | - | - | - |
|  | _100_GWGNGCGLF_108_ | A24 | - | - | - | - |
|  |  |  |  |  |  |  |
| NS1 | _12_ELKCGSGIF_20_ |  |  |  |  |  |
|  | _12_ELKCGSGIF_20_ | A26, B8, B62 | - | - | - | - |
|  | _25_VHTWTEQYKFQ_35_ |  |  |  |  |  |
|  | _26_HTWTEQYKF_34_ | A1, A24, A26, B8, B27, B58, B62 | - | - | - | - |
|  | _193_AVHADMGYWIES_204_ |  |  |  |  |  |
|  | _193_AVHADMGYW_201_ | A26, B58 | - | - | - | - |
|  | _194_VHADMGYWI_202_ | A24, B39 | - | - | DR | - |
|  | _195_HADMGYWIE_203_ | A1 | - | - | - | - |
|  | _229_HTLWSNGVLES_239_ |  |  |  |  |  |
|  | _229_HTLWSNGVL_237_ | A1, B8, B39, B62 | - | - | - | - |
|  | _231_LWSNGVLES_239_ | - | - | - | - | DR |
|  | _325_GEDGCWYGMEIRP_337_ |  |  |  |  |  |
|  | _325_GEDGCWYGM_333_ | B44 | - | - | - | - |
|  | _328_GCWYGMEIR_336_ | - | - | A3 | - | - |
|  |  |  |  |  |  |  |
| NS3 | _46_FHTMWHVTRG_55_ |  |  |  |  |  |
|  | _46_FHTMWHVTR_54_ | B39 | A3 | - | DR | - |
|  | _47_HTMWHVTRG_55_ | - | A3 | - | - | - |
|  | _189_LTIMDLHPG_197_ |  |  |  |  |  |
|  | _189_LTIMDLHPG_197_ | - | - | - | **DR** | **DR** |
|  | _256_EIVDLMCHATFT_267_ |  |  |  |  |  |
|  | _256_EIVDLMCHA_264_ | A26 | A2 | - | - | - |
|  | _257_IVDLMCHAT_265_ | - | - | - | DR | - |
|  | _258_VDLMCHATF_266_ | B8, **B44** | - | **B44** | DR | - |
|  | _259_DLMCHATFT_267_ | - | **A2** | **A2** | - | - |
|  | _296_AARGYISTRV_305_ |  |  |  |  |  |
|  | _296_AARGYISTR_304_ | **A3** | **A3** | **A3** | - | - |
|  | _297_ARGYISTRV_305_ | B27 | - | - | - | - |
|  | _313_IFMTATPPG_321_ |  |  |  |  |  |
|  | _313_IFMTATPPG_321_ | - | - | - | **DR** | **DR** |
|  | _357_GKTVWFVPSIK_367_ |  |  |  |  |  |
|  | _358_KTVWFVPSI_366_ | **A2**, A24, A26, B58 | **A2** | **A2** | - | - |
|  | _359_TVWFVPSIK_367_ | **A3** | **A3** | **A3** | - | - |
|  | _383_VIQLSRKTFD_392_ |  |  |  |  |  |
|  | _383_VIQLSRKTF_391_ | B7, B8, B62 | - | - | DR | - |
|  | _384_IQLSRKTFD_392_ | - | - | - | DR | - |
|  | _406_VVTTDISEMGANF_418_ |  |  |  |  |  |
|  | _406_VVTTDISEM_414_ | A26, B62 | - | - | DR | - |
|  | _407_VTTDISEMG_415_ | - | - | - | DR | - |
|  | _408_TTDISEMGA_416_ | A1 | - | - | - | - |
|  | _410_DISEMGANF_418_ | A1, A26, B62 | - | - | - | - |
|  | _537_LMRRGDLPVWL_547_ |  |  |  |  |  |
|  | _537_LMRRGDLPV_545_ | **A2**, B8, B62 | - | **A2** | DR | - |
|  | _538_MRRGDLPVW_546_ | B27 | - | - | - | - |
|  | _539_RRGDLPVWL_547_ | B27, B39 | - | - | - | - |
|  |  |  |  |  |  |  |
| NS4a | _126_QRTPQDNQL_134_ |  |  |  |  |  |
|  | _126_QRTPQDNQL_134_ | B27, B39 | - | - | - | - |
|  |  |  |  |  |  |  |
| NS4b | _35_PASAWTLYAVATT_47_ |  |  |  |  |  |
|  | _36_ASAWTLYAV_44_ | A1, **A2** | **A2** | **A2** | - | - |
|  | _37_SAWTLYAVA_45_ | - | **A2** | **A2** | - | - |
|  | _39_WTLYAVATT_47_ | - | **A2** | **A2** | DR | - |
|  | _118_HYAIIGPGLQAKATREAQKR_137_ |  |  |  |  |  |
|  | _118_HYAIIGPGL_126_ | A24, B39 | - | - | - | - |
|  | _119_YAIIGPGLQ_127_ | - | - | - | **DR** | **DR** |
|  | _120_AIIGPGLQA_128_ | - | A3 | - | - | - |
|  | _121_IIGPGLQAK_129_ | **A3** | **A3** | - | - | - |
|  | _126_LQAKATREA_134_ | B62 | - | A2 | DR | - |
|  | _127_QAKATREAQ_135_ | B8 | - | - | - | - |
|  | _128_AKATREAQK_136_ | B27 | A3 | - | - | - |
|  | _129_KATREAQKR_137_ | - | A3 | - | - | - |
|  | _139_AAGIMKNPTVDGI_151_ |  |  |  |  |  |
|  | _142_IMKNPTVDG_150_ | - | A3 | - | DR | - |
|  | _143_MKNPTVDGI_151_ | - | - | B7 | DR | - |
|  | _223_ANIFRGSYLAGAGL_236_ |  |  |  |  |  |
|  | _223_ANIFRGSYL_231_ | B7 | - | A2 | - | - |
|  | _224_NIFRGSYLA_232_ | **A2** | **A3** | **A2, A3** | - | - |
|  | _225_IFRGSYLAG_233_ | - | - | - | DR | - |
|  | _226_FRGSYLAGA_234_ | B27 | **A2** | **A2** | **DR** | **DR** |
|  | _228_GSYLAGAGL_236_ | B39, B44, B62 | - | - | - | - |
|  |  |  |  |  |  |  |
| NS5 | _6_GETLGEKWK_14_ |  |  |  |  |  |
|  | _6_GETLGEKWK_14_ | B44 | - | - | - | - |
|  | _79_DLGCGRGGWSYY_90_ |  |  |  |  |  |
|  | _81_GCGRGGWSY_89_ | A1, B62 | - | - | - | - |
|  | _82_CGRGGWSYY_90_ | A1, A26, B62 | - | - | - | - |
|  | _141_DTLLCDIGESS_151_ |  |  |  |  |  |
|  | _142_TLLCDIGES_150_ | - | - | A2 | - | - |
|  | _143_LLCDIGESS_151_ | - | - | - | DR | - |
|  | _209_PLSRNSTHEMYW_220_ |  |  |  |  |  |
|  | _210_LSRNSTHEM_218_ | **B7**, B58, B62 | - | **B7** | DR | - |
|  | _211_SRNSTHEMY_219_ | A1, B8, B27 | - | - | - | - |
|  | _212_RNSTHEMYW_220_ | B58 | - | - | - | - |
|  | _342_AMTDTTPFGQQRVFKEKVDTRT_363_ |  |  |  |  |  |
|  | _343_MTDTTPFGQ_351_ | A1 | - | - | - | - |
|  | _345_DTTPFGQQR_353_ | - | **A3** | **A3** | - | - |
|  | _346_TTPFGQQRV_354_ | A1, A26 | - | - | - | - |
|  | _347_TPFGQQRVF_355_ | **B7**, B8 | - | **B7** | - | - |
|  | _348_PFGQQRVFK_356_ | - | A3 | - | - | - |
|  | _349_FGQQRVFKE_357_ | - | - | - | DR | - |
|  | _350_GQQRVFKEK_358_ | **A3**, B27 | **A3** | - | - | - |
|  | _354_VFKEKVDTR_362_ | - | A3 | - | - | - |
|  | _450_CVYNMMGKREKKLGEFG_466_ |  |  |  |  |  |
|  | _450_CVYNMMGKR_458_ | **A3** | **A3** | **A3** | - | - |
|  | _451_VYNMMGKRE_459_ | - | - | - | DR | - |
|  | _452_YNMMGKREK_460_ | - | **A3** | **A3** | **DR** | **DR** |
|  | _453_NMMGKREKK_461_ | **A3** | **A3** | **A3** | - | - |
|  | _454_MMGKREKKL_462_ | B8 | A2 | - | - | - |
|  | _457_KREKKLGEF_465_ | A1, B8, B27 | - | - | - | - |
|  | _458_REKKLGEFG_466_ | - | - | B44 | - | - |
|  | _468_AKGSRAIWYMWLGAR_482_ |  |  |  |  |  |
|  | _469_KGSRAIWYM_477_ | B58 | - | - | - | - |
|  | _470_GSRAIWYMW_478_ | B58 | - | - | - | - |
|  | _471_SRAIWYMWL_479_ | B27, B39 | - | - | - | - |
|  | _473_AIWYMWLGA_481_ | **A2** | - | **A2** | - | - |
|  | _474_IWYMWLGAR_482_ | - | - | - | DR | - |
|  | _531_YADDTAGWDTRIT_543_ |  |  |  |  |  |
|  | _531_YADDTAGWD_539_ | - | - | - | DR | - |
|  | _534_DTAGWDTRI_542_ | A1, A26 | A2 | - | - | - |
|  | _568_IFKLTYQNKVV_578_ |  |  |  |  |  |
|  | _568_IFKLTYQNK_576_ | A3, A24 | - | - | - | - |
|  | _569_FKLTYQNKV_577_ | **A2** | **A2** | **A2** | DR | - |
|  | _570_KLTYQNKVV_578_ | **A2** | **A2** | - | - | - |
|  | _597_DQRGSGQVGTYGLNTFTNME_616_ |  |  |  |  |  |
|  | _599_RGSGQVGTY_607_ | A1, B58, B62 | - | - | - | - |
|  | _601_SGQVGTYGL_609_ | B39 | - | - | - | - |
|  | _604_VGTYGLNTF_612_ | B58, B62 | - | - | DR | - |
|  | _605_GTYGLNTFT_613_ | - | - | A2 | - | - |
|  | _606_TYGLNTFTN_614_ | A24 | - | - | - | - |
|  | _607_YGLNTFTNM_615_ | A26 | - | - | DR | - |
|  | _658_RMAISGDDCVVKP_670_ |  |  |  |  |  |
|  | _659_MAISGDDCV_667_ | - | - | A2, B7 | - | - |
|  | _660_AISGDDCVV_668_ | **A2** | **A2** | **A2** | - | - |
|  | _661_ISGDDCVVK_669_ | **A3** | **A3** | - | - | - |
|  | _707_VPFCSHHFH_715_ |  |  |  |  |  |
|  | _707_VPFCSHHFH_715_ | - | - | A3 | DR | - |
|  | _765_LMYFHRRDLRLA_776_ |  |  |  |  |  |
|  | _765_LMYFHRRDL_773_ | B39, B8, B62 | - | - | **DR** | **DR** |
|  | _766_MYFHRRDLR_774_ | **A3** | **A3** | **A3** | DR | - |
|  | _767_YFHRRDLRL_775_ | A1, A24, B8, B39 | A2 | - | DR | - |
|  | _768_FHRRDLRLA_776_ | - | - | - | DR | - |
|  | _790_PTSRTTWSIHA_800_ |  |  |  |  |  |
|  | _790_PTSRTTWSI_798_ | A1, A24 | A2 | - | - | - |
|  | _792_SRTTWSIHA_800_ | B27 | - | - | - | - |
|  |  |  |  |  |  |  |

^a^ Amino acid positions of the pan-DENV sequences and the predicted nonamers are numbered according to the sequence alignments of the 4 DENV types

^b^ HLA supertype-restrictions that were predicted by at least two prediction models are highlighted in bold

^c^ Peptides specific to HLA class I supertypes were predicted by use of NetCTL (A1, A2, A3, A24, A26, B7, B8, B27, B39, B44, B58

and B62), ARB (A2, A3, B44, and B7) and Multipred (A2 and A3)

^d^ Sequences identified as specific to class II were predicted by use of TEPITOPE (DR) and Multipred (DR) as described in methods
