# Supplementary material for: Conservation and Variability of Dengue Virus Proteins: Implications for Vaccine Design
Source: PLoS Negl Trop Dis. 2008 Aug 13;2(8):e272. doi: 10.1371/journal.pntd.0000272 (PMC2491585; doi:10.1371/journal.pntd.0000272)
Supplement: Table S6 — Intra-type representation of candidate putative HLA supertype-restricted nonamer peptides predicted by immunoinformatics algorithms. (0.22 MB DOC) [file pntd.0000272.s008.doc]

| DENV  protein | Pan-DENV sequence and the putative HLA supertype-restricted nonamer peptide(s)^a^ | Intra-type representation (%)^b^ and total sequences analyzed (#)^c^ | | | |
| --- | --- | --- | --- | --- | --- |
|  |  | DENV-1 | DENV-2 | DENV-3 | DENV-4 |
|  |  |  |  |  |  |
| E | _97_VDRGWGNGCGLFGKG_111_ |  |  |  |  |
|  | _99_RGWGNGCGL_107_ | 98%(580) | 98%(811) | 100%(372) | 100%(320) |
|  | _100_GWGNGCGLF_108_ | 99%(580) | 98%(811) | 100%(372) | 95%(320) |
|  |  |  |  |  |  |
| NS1 | _12_ELKCGSGIF_20_ |  |  |  |  |
|  | _12_ELKCGSGIF_20_ | 99%(366) | 95%(603) | 83%(201) | 99%(141) |
|  | _25_VHTWTEQYKFQ_35_ |  |  |  |  |
|  | _26_HTWTEQYKF_34_ | 99%(350) | 96%(555) | 100%(197) | 95%(140) |
|  | _193_AVHADMGYWIES_204_ |  |  |  |  |
|  | _193_AVHADMGYW_201_ | 100%(104) | 95%(197) | 98%(117) | 100%(28) |
|  | _194_VHADMGYWI_202_ | 100%(104) | 96%(197) | 98%(117) | 96%(28) |
|  | _195_HADMGYWIE_203_ | 100%(104) | 97%(197) | 98%(117) | 96%(28) |
|  | _229_HTLWSNGVLES_239_ |  |  |  |  |
|  | _229_HTLWSNGVL_237_ | 98%(124) | 97%(215) | 97%(117) | 100%(28) |
|  | _231_LWSNGVLES_239_ | 97%(126) | 97%(215) | 98%(117) | 100%(28) |
|  | _325_GEDGCWYGMEIRP_337_ |  |  |  |  |
|  | _325_GEDGCWYGM_333_ | 98%(112) | 98%(202) | 98%(113) | 100%(28) |
|  | _328_GCWYGMEIR_336_ | 100% (104) | 99%(197) | 100%(115) | 100%(28) |
|  |  |  |  |  |  |
| NS3 | _46_FHTMWHVTRG_55_ |  |  |  |  |
|  | _46_FHTMWHVTR_54_ | 100%(89) | 100%(132) | 100%(68) | 100%(28) |
|  | _47_HTMWHVTRG_55_ | 100%(89) | 100%(132) | 100%(68) | 100%(28) |
|  | _189_LTIMDLHPG_197_ |  |  |  |  |
|  | _189_LTIMDLHPG_197_ | 100%(97) | 99%(141) | 100%(97) | 100%(29) |
|  | _256_EIVDLMCHATFT_267_ |  |  |  |  |
|  | _256_EIVDLMCHA_264_ | 99%(98) | 100%(137) | 100%(103) | 100%(29) |
|  | _257_IVDLMCHAT_265_ | 99%(98) | 100%(137) | 100%(103) | 100%(29) |
|  | _258_VDLMCHATF_266_ | 99%(98) | 100%(137) | 100%(103) | 100%(29) |
|  | _259_DLMCHATFT_267_ | 99%(98) | 100%(137) | 100%(103) | 100%(29) |
|  | _296_AARGYISTRV_305_ |  |  |  |  |
|  | _296_AARGYISTR_304_ | 97%(90) | 100%(135) | 97%(74) | 100%(27) |
|  | _297_ARGYISTRV_305_ | 97%(90) | 100%(135) | 97%(74) | 100%(27) |
|  | _313_IFMTATPPG_321_ |  |  |  |  |
|  | _313_IFMTATPPG_321_ | 100%(90) | 100%(135) | 100%(74) | 100%(27) |
|  | _357_GKTVWFVPSIK_367_ |  |  |  |  |
|  | _358_KTVWFVPSI_366_ | 99%(90) | 100%(135) | 99%(181) | 96%(27) |
|  | _359_TVWFVPSIK_367_ | 99%(90) | 100%(135) | 100%(181) | 100%(27) |
|  | _383_VIQLSRKTFD_392_ |  |  |  |  |
|  | _383_VIQLSRKTF_391_ | 81%(90) | 99%(135) | 99%(181) | 100%(27) |
|  | _384_IQLSRKTFD_392_ | 81%(90) | 99%(135) | 98%(181) | 100%(27) |
|  | _406_VVTTDISEMGANF_418_ |  |  |  |  |
|  | _406_VVTTDISEM_414_ | 98%(90) | 99%(135) | 98%(181) | 100%(27) |
|  | _407_VTTDISEMG_415_ | 98%(90) | 99%(135) | 99%(181) | 100%(27) |
|  | _408_TTDISEMGA_416_ | 98%(90) | 100%(135) | 99%(181) | 100%(27) |
|  | _410_DISEMGANF_418_ | 98%(90) | 100%(135) | 99%(181) | 100%(27) |
|  | _537_LMRRGDLPVWL_547_ |  |  |  |  |
|  | _537_LMRRGDLPV_545_ | 99%(89) | 100%(133) | 99%(181) | 93%(27) |
|  | _538_MRRGDLPVW_546_ | 99%(89) | 100%(133) | 99%(181) | 93%(27) |
|  | _539_RRGDLPVWL_547_ | 100%(89) | 100%(133) | 99%(181) | 93%(27) |
|  |  |  |  |  |  |
| NS4a | _126_QRTPQDNQL_134_ |  |  |  |  |
|  | _126_QRTPQDNQL_134_ | 98%(87) | 100%(126) | 100%(70) | 100%(26) |
|  |  |  |  |  |  |
| NS4b | _35_PASAWTLYAVATT_47_ |  |  |  |  |
|  | _36_ASAWTLYAV_44_ | 100%(89) | 100%(127) | 100%(70) | 100%(27) |
|  | _37_SAWTLYAVA_45_ | 100%(89) | 100%(127) | 100%(70) | 100%(27) |
|  | _39_WTLYAVATT_47_ | 100%(89) | 100%(127) | 100%(70) | 100%(27) |
|  | _118_HYAIIGPGLQAKATREAQKR_137_ |  |  |  |  |
|  | _118_HYAIIGPGL_126_ | 100%(89) | 97%(127) | 100%(70) | 98%(109) |
|  | _119_YAIIGPGLQ_127_ | 100%(89) | 97%(127) | 100%(70) | 98%(109) |
|  | _120_AIIGPGLQA_128_ | 100%(89) | 97%(127) | 100%(70) | 98%(109) |
|  | _121_IIGPGLQAK_129_ | 100%(89) | 97%(127) | 100%(70) | 99%(109) |
|  | _126_LQAKATREA_134_ | 99%(89) | 97%(127) | 100%(70) | 100%(109) |
|  | _127_QAKATREAQ_135_ | 99%(89) | 95%(127) | 100%(70) | 100%(109) |
|  | _128_AKATREAQK_136_ | 99%(89) | 95%(127) | 100%(70) | 100%(109) |
|  | _129_KATREAQKR_137_ | 99%(89) | 95%(127) | 100%(70) | 100%(109) |
|  | _139_AAGIMKNPTVDGI_151_ |  |  |  |  |
|  | _142_IMKNPTVDG_150_ | 96%(89) | 98%(127) | 97%(70) | 100%(109) |
|  | _143_MKNPTVDGI_151_ | 98%(89) | 98%(127) | 97%(70) | 100%(109) |
|  | _223_ANIFRGSYLAGAGL_236_ |  |  |  |  |
|  | _223_ANIFRGSYL_231_ | 100%(87) | 100%(129) | 100%(70) | 98%(109) |
|  | _224_NIFRGSYLA_232_ | 100%(87) | 100%(129) | 100%(70) | 100%(109) |
|  | _225_IFRGSYLAG_233_ | 100%(87) | 100%(129) | 100%(70) | 100%(109) |
|  | _226_FRGSYLAGA_234_ | 100%(87) | 100%(129) | 97%(70) | 100%(109) |
|  | _228_GSYLAGAGL_236_ | 100%(87) | 100%(129) | 97%(70) | 100%(109) |
|  |  |  |  |  |  |
| NS5 | _6_GETLGEKWK_14_ |  |  |  |  |
|  | _6_GETLGEKWK_14_ | 92%(87) | 98%(131) | 100%(70) | 100%(109) |
|  | _79_DLGCGRGGWSYY_90_ |  |  |  |  |
|  | _81_GCGRGGWSY_89_ | 100%(87) | 98%(130) | 100%(78) | 100%(27) |
|  | _82_CGRGGWSYY_90_ | 100%(87) | 98%(130) | 100%(80) | 100%(27) |
|  | _141_DTLLCDIGESS_151_ |  |  |  |  |
|  | _142_TLLCDIGES_150_ | 100%(87) | 100%(130) | 100%(74) | 100%(27) |
|  | _143_LLCDIGESS_151_ | 100%(87) | 100%(130) | 100%(74) | 100%(27) |
|  | _209_PLSRNSTHEMYW_220_ |  |  |  |  |
|  | _210_LSRNSTHEM_218_ | 100%(87) | 100%(130) | 100%(70) | 100%(27) |
|  | _211_SRNSTHEMY_219_ | 100%(87) | 100%(130) | 100%(70) | 100%(27) |
|  | _212_RNSTHEMYW_220_ | 100%(87) | 100%(130) | 100%(70) | 100%(27) |
|  | _342_AMTDTTPFGQQRVFKEKVDTRT_363_ |  |  |  |  |
|  | _343_MTDTTPFGQ_351_ | 100%(87) | 98%(126) | 99%(165) | 96%(27) |
|  | _345_DTTPFGQQR_353_ | 100%(87) | 100%(126) | 100%(165) | 96%(27) |
|  | _346_TTPFGQQRV_354_ | 100%(87) | 100%(126) | 100%(165) | 96%(27) |
|  | _347_TPFGQQRVF_355_ | 100%(87) | 100%(126) | 100%(157) | 96%(27) |
|  | _348_PFGQQRVFK_356_ | 100%(87) | 100%(126) | 100%(157) | 96%(27) |
|  | _349_FGQQRVFKE_357_ | 100%(87) | 100%(126) | 100%(157) | 100%(27) |
|  | _350_GQQRVFKEK_358_ | 100%(87) | 100%(126) | 99%(157) | 100%(27) |
|  | _354_VFKEKVDTR_362_ | 100%(87) | 100%(126) | 99%(157) | 100%(27) |
|  | _450_CVYNMMGKREKKLGEFG_466_ |  |  |  |  |
|  | _450_CVYNMMGKR_458_ | 100%(87) | 95%(126) | 97%(155) | 100%(27) |
|  | _451_VYNMMGKRE_459_ | 100%(87) | 95%(126) | 97%(155) | 100%(27) |
|  | _452_YNMMGKREK_460_ | 100%(87) | 97%(126) | 97%(155) | 100%(27) |
|  | _453_NMMGKREKK_461_ | 100%(87) | 97%(126) | 97%(155) | 100%(27) |
|  | _454_MMGKREKKL_462_ | 100%(87) | 94%(126) | 97%(155) | 100%(27) |
|  | _457_KREKKLGEF_465_ | 100%(87) | 97%(126) | 99%(155) | 100%(27) |
|  | _458_REKKLGEFG_466_ | 100%(87) | 97%(126) | 99%(155) | 100%(27) |
|  | _468_AKGSRAIWYMWLGAR_482_ |  |  |  |  |
|  | _469_KGSRAIWYM_477_ | 98%(87) | 99%(126) | 99%(155) | 100%(27) |
|  | _470_GSRAIWYMW_478_ | 98%(87) | 100%(128) | 99%(155) | 100%(27) |
|  | _471_SRAIWYMWL_479_ | 98%(87) | 100%(128) | 100%(155) | 100%(27) |
|  | _473_AIWYMWLGA_481_ | 98%(87) | 100%(128) | 99%(157) | 100%(27) |
|  | _474_IWYMWLGAR_482_ | 97%(87) | 100%(128) | 99%(157) | 100%(27) |
|  | _531_YADDTAGWDTRIT_543_ |  |  |  |  |
|  | _531_YADDTAGWD_539_ | 100%(87) | 100%(128) | 99%(157) | 100%(27) |
|  | _534_DTAGWDTRI_542_ | 100%(87) | 100%(128) | 99%(157) | 100%(27) |
|  | _568_IFKLTYQNKVV_578_ |  |  |  |  |
|  | _568_IFKLTYQNK_576_ | 100%(87) | 98%(130) | 97%(159) | 100%(27) |
|  | _569_FKLTYQNKV_577_ | 100%(87) | 97%(130) | 98%(159) | 100%(27) |
|  | _570_KLTYQNKVV_578_ | 100%(87) | 97%(130) | 98%(159) | 100%(27) |
|  | _597_DQRGSGQVGTYGLNTFTNME_616_ |  |  |  |  |
|  | _599_RGSGQVGTY_607_ | 100%(87) | 88%(130) | 98%(159) | 100%(27) |
|  | _601_SGQVGTYGL_609_ | 100%(87) | 88%(130) | 98%(159) | 100%(27) |
|  | _604_VGTYGLNTF_612_ | 100%(87) | 88%(130) | 97%(159) | 100%(27) |
|  | _605_GTYGLNTFT_613_ | 100%(87) | 88%(114) | 99%(158) | 100%(27) |
|  | _606_TYGLNTFTN_614_ | 100%(87) | 98%(130) | 99%(159) | 100%(27) |
|  | _607_YGLNTFTNM_615_ | 98%(87) | 98%(130) | 99%(159) | 100%(27) |
|  | _658_RMAISGDDCVVKP_670_ |  |  |  |  |
|  | _659_MAISGDDCV_667_ | 100%(87) | 100%(130) | 97%(159) | 100%(27) |
|  | _660_AISGDDCVV_668_ | 100%(87) | 100%(130) | 97%(159) | 100%(27) |
|  | _661_ISGDDCVVK_669_ | 100%(87) | 100%(130) | 97%(159) | 100%(27) |
|  | _707_VPFCSHHFH_715_ |  |  |  |  |
|  | _707_VPFCSHHFH_715_ | 98%(87) | 100%(130) | 100%(159) | 96%(27) |
|  | _765_LMYFHRRDLRLA_776_ |  |  |  |  |
|  | _765_LMYFHRRDL_773_ | 100%(87) | 98%(130) | 99%(159) | 100%(27) |
|  | _766_MYFHRRDLR_774_ | 100%(87) | 98%(130) | 99%(159) | 100%(27) |
|  | _767_YFHRRDLRL_775_ | 100%(87) | 98%(130) | 99%(159) | 100%(27) |
|  | _768_FHRRDLRLA_776_ | 100%(87) | 98%(130) | 98%(159) | 100%(27) |
|  | _790_PTSRTTWSIHA_800_ |  |  |  |  |
|  | _790_PTSRTTWSI_798_ | 99%(87) | 98%(128) | 98%(157) | 100%(27) |
|  | _792_SRTTWSIHA_800_ | 99%(87) | 98%(128) | 100%(157) | 100%(27) |
|  |  |  |  |  |  |

^a^ Amino acid positions of the pan-DENV sequences and the predicted nonamers are numbered according to the sequence alignments of the 4 DENV types

^b^ Rounded to whole number

^c^ The total number of sequences analyzed (2005 dataset) may not match the total number of sequences collected for each protein because both partial and full-length sequences were used for the sequence alignments, with the results that some regions have more sequence information than others
