# Supplementary material for: Conservation and Variability of Dengue Virus Proteins: Implications for Vaccine Design
Source: PLoS Negl Trop Dis. 2008 Aug 13;2(8):e272. doi: 10.1371/journal.pntd.0000272 (PMC2491585; doi:10.1371/journal.pntd.0000272)
Supplement: Alternative Language Abstract S1 — Translation of the abstract into Chinese by Guang Lan Zhang. (0.06 MB PDF) [file pntd.0000272.s010.pdf]

登革病毒蛋白的保守及变异对疫苗设计的影响和意义

Asif M. Khan[1], Olivo Miotto[1,2], Eduardo J.M. Nascimento[3], K.N. Srinivasan[4,5], A.T. Heiny[1], Guang Lan Zhang[6], E.T. Marques[3,4], Tin Wee Tan[1], Vladimir Brusic[6], Jerome Salmon[4], J. Thomas August[4*]

Running Title Head: 登革病毒蛋白的保守序列

[1]Department of Biochemistry, Yong Loo Lin School of Medicine, National University of Singapore, 8 Medical Drive, Singapore 117597, Singapore

[2]Institute of Systems Science, National University of Singapore, 25 Heng Mui Keng Terrace, Singapore 119615, Singapore

[3]Department of Medicine, Division of Infectious Diseases, The Johns Hopkins University School of Medicine, 725 North Wolfe Street, Baltimore, MD 21205, United States of America

[4]Department of Pharmacology and Molecular Sciences, The Johns Hopkins University School of Medicine, 725 North Wolfe Street, Baltimore, MD 21205, United States of America

[5]Product Evaluation & Registration Division, Centre for Drug Administration, Health Sciences Authority, 11 Biopolis Way, Singapore 138667, Singapore

[6]Cancer Vaccine Center, Dana-Farber Cancer Institute, 77 Avenue Louis Pasteur, HIM 418 Boston, MA 02115, United States of America

*Corresponding author.
E-mail: taugust@jhmi.edu
Phone: +1 410 955 8484
Fax: +1 410 502 3066

摘要

RNA 病毒的典型特征, 基因差异和快速进化, 是由于主体的免疫反应导致 RNA 复制的高变异率以及对增强病毒环境适应能力的变异的选择所导致的。基因差异在基因组的分布是不均匀的，这是因为那些会对病毒的存活产生有害效应的变异受到高度限制。因此一些 RNA 病毒蛋白位点存在多个变异，而那些对病毒结构功能必需的位点则在进化上高度保守稳定。确定这些重要位点的位置和它们的演化对多项应用， 包括疾病的诊断以及预防和治疗， 都有重要意义。

方法/主要发现

本文报告了一个通过大规模分析登革病毒蛋白组以确定进化上高度保守的蛋白序列的方法.。因为多数 T 细胞表位是九肽，我们的方法是从九肽着手。我们分别于 2005 年和 2007 年从 NCBI Entrez 蛋白数据库收集了 9,512 条和 12,404 条登革病毒蛋白序列。分析结果确定了 44 条保守序列(泛登革病毒蛋白序列), 其中多数属于非结构蛋白。这些序列在 80%以上的已知登革病毒蛋白序列中是保守的，它们的长度总和相当于登革病毒多蛋白长度的 15%。34 条(~77%)泛登革病毒蛋白序列存在于 95%以上的登革病毒各种血清型序列中，其中 27 条(~61%)序列在其他黄病毒蛋白序列中也呈现保守。泛登革病毒蛋白序列中，变异发生率很低(0%-5%)，而在非泛登革病毒蛋白序列中，序列变异发生率是 60%到 85%。进一步研究表明这些保守序列大多具有免疫相关性，其中 34 条序列包含多个预测的人白细胞抗原超型结合肽，26 条序列包含由转基因鼠研究确定的 T 细胞表位或由其他试验表明对人体有免疫原性。

结论/意义

含 9 个及以上氨基酸的 44 条泛登革病毒蛋白序列高度保守。它们在 80%以上的已知登革病毒蛋白序列中呈现保守，并且其中多数与已经鉴定或报告的 T 细胞表位和大量预测的人白细胞抗原超型结合肽有免疫相关性。这些序列在整个登革病毒基因进化过程呈现保守的事实表明它们可能对疾病诊断以及预防和治疗都起到作用。本文所描述的生物信息学和传统试验相结合的方法为大规模系统分析病毒的保守序列，特别是快速变异的病毒，如甲型流感病毒和人类免疫缺陷病毒，搭建了一个新的平台。
